# Marine mammals as models for charting the evolution of social vocal rhythm

**DOI:** 10.1186/s12915-026-02675-8

**Published:** 2026-07-04

**Authors:** Peter F. Cook, Sophie Flem, Peter T. Madsen, Andrea Ravignani, Pete Gremore, Carson Hood, Yannick Jadoul, Danai Papageorgiou, Michael Pedersen, Teresa Raimondi, Andrew Rouse, Pernille M. Sørensen, Simone Videsen, Stephanie L. King

**Affiliations:** 1 Master’s in Marine Mammal Science, New College of Florida, Sarasota, USA; 2 Institute for Marine Sciences, University of California Santa Cruz, Santa Cruz, USA; 3 Department of Human Neurosciences, Sapienza University of Rome, Rome, Italy; 4 Research Center of Neuroscience “CRiN-Daniel Bovet”, Sapienza University of Rome, Rome, Italy; 5 Center for Music in the Brain, Department of Clinical Medicine, Aarhus University & The Royal Academy of Music Aarhus/Aalborg, Aalborg, Denmark; 6 School of Biological Sciences, University of Bristol, Bristol, BS8 1TQ UK; 7 Department of Evolutionary Anthropology, University of Zurich, Zurich, CH-8057 Switzerland; 8 Marine Bioacoustics Lab, Department of Biology, Aarhus University, Aarhus, 8000 Denmark

**Keywords:** Marine mammals, Cetaceans, Pinnipeds, Vocal behaviour, Communication, Social behaviour, Rhythm, Synchrony, Vocal physiology, Vocal neurobiology

## Abstract

In this Perspective, we suggest combining physiological, neurobiological, behavioural, and social data from multiple marine mammal species to create evolutionary models of the emergence of social vocal coordination. Marine mammal sound production mechanisms have evolved to manage vocalizing and breathing in an aquatic environment, potentially releasing functional constraints on the control of vocal timing. Intervals in rhythmic cetacean vocalizations cover a remarkable temporal range, from less than a millisecond in porpoise burst pulses to 10 s in sperm whale slow clicks. Many cetaceans demonstrate temporally coordinated social behaviour, while pinnipeds express high variability in vocal plasticity and social behaviour. The systematic variability of vocal production mechanisms, vocal rhythms, and sociality can be compared phylogenetically to generate models for the evolution of social rhythm in the vocal domain.

**Supplementary Information:**

The online version contains supplementary material available at 10.1186/s12915-026-02675-8.

## Framing the evolution of social rhythm

In social contexts, many species produce vocalizations that are rhythmic—that is, structured in time. This rhythmicity may relate to the social function of the calls in a variety of ways, carrying information about the caller’s physiological state or fitness. In some cases, rhythmic calls may also be temporally coordinated with the vocalizations of conspecifics; in other words, the timing of an individual’s calls may depend on the timing of its conspecifics’ calls. What is the adaptive value of these social vocal rhythms? And how did the mechanisms supporting them evolve? In this Perspective, we propose the value of a multi-clade, integrative analysis of marine mammals towards answering these questions.

To date, research on mammalian social rhythm has primarily focused on humans, who produce a wide range of complex, temporally organized social vocal behaviour [1–5]. In humans, temporal coordination of behaviour, that is, co-timing, serves both a coordinating role and an affiliative one. The precise timing of turn taking during human conversation, on the one hand, and synchrony (defined here as production of in-phase, temporally overlapping rhythmic signals by two or more conspecifics) in song, on the other hand, showcase how humans can organize complex behaviour through shared auditory rhythms [6, 7]. In turn, co-timing drives increased positive emotion towards conspecifics [8]. Humans who coordinate their behaviour in time reliably report increased positive attitudes towards their timing partners across a range of experimental and natural scenarios.

Speech and music are among the most complex social artefacts in the animal kingdom, and both have been suggested to be unique to humans. However, humans are not the only social species that rely on vocal coordination in social contexts. Outside of humans, social co-timing has been most frequently studied in phylogenetically distant species such as chorusing insects and amphibians [9]. Clearly, rhythm serves broadly conserved evolutionary functions in animal social behaviour. Shared, predictable signals are a temporal framework for social coordination. Proximally, behaviour, including production of vocal signals, is produced by the nervous system, and rhythm is also the fundamental organizing principle of neuronal activity, allowing coordinated activity across distributed sensors and effectors. From cellular coordination to inter-individual coordination, rhythm is the framework that makes it possible. Are apparently complex and unique human social behaviours like language and song just expressions of the same broadly conserved mechanisms driving cricket chorusing? We argue not. To clarify why, we must distinguish between obligate and volitional behaviour.

Obligate behaviours are reliably produced given specific triggering conditions, while volitional behaviours are produced or not dependent on an animal’s cognitive state. This distinction is intuitive in humans if one contrasts “reflexive” behaviour, such as knee extension following patellar impact from a doctor’s mallet, with “deliberative” behaviour, such as deciding which college to attend. However, in practice, most animal behaviour falls on a continuum from more to less automatic. For simplicity, we use the term volitional in this manuscript to refer to motor output that is driven primarily by top-down nervous-system signaling generated above primary effector control systems. Such signaling is distinctive for not always and only being generated in specific environmental circumstances, and, in chordates, depends on descending signals from association cortex (see [10]). In contrast, obligate behaviour can be reflexive or triggered by specific subcortical mechanisms. Strong evidence in insects and amphibians shows that, unlike in humans, the temporal coordination of their acoustic signals is obligate.

The temporally coordinated social vocal rhythms of most insects and anurans are similar to obligate biorhythms in diverse taxa. The rhythms of locomotion, respiration, and circulation in most species are driven by endogenous pacemakers and coupling between bodies and environments [11, 12]. Likewise, the coordination of vocalizations in lower phyla commonly involves pacemaker-driven output that can be temporarily suppressed or facilitated in direct response to receiving a signal from a conspecific [13, 14]. Resultant temporal coordination is mechanistically reactive and domain limited. Importantly, there are many different modes of temporal coordination and entrainment [15]. The most exacting form of temporal coordination observed in animal behaviour is synchrony, defined as precise co-occurrence of two rhythmically matched signals or behaviours over time. Certain species of fireflies and crickets do dynamically and precisely alter their signal rate to synchronize with their neighbours, and by so-doing they better advertise their presence for mating. It has been suggested [14] that this might functionally parallel early hominid vocal (and possibly drumming) synchrony in transmitting group location over distance. But even in cases where broad evolutionary functions of synchronous signaling may be shared, mechanisms may be very different. Nearly all invertebrates and amphibians that entrain or synchronize their calling or signaling do so always and only under very specific social and environmental circumstances.

In contrast to insects and amphibians, much of human social entrainment and synchrony, including vocal coordination in speech and song, is volitional. Humans have flexible control over their vocal behaviour, including timing [16, 17]. Most mammals have some capability for volitionally initiating or inhibiting a species-typical call [18], but few with the ease and precision shown by humans. Human facility with vocal timing allows creation and coordination of complex rhythmic structures. In addition, humans can choose whether or not to synchronize motor and vocal behaviour with external signals—there is evidence that humans are less likely to entrain their behaviour to that of people with whom they are less closely affiliated [19]. This suggests that, while the core function of rhythm in both cooperative and competitive social coordination is evolutionarily conserved (i.e., it provides a mutually accesible temporal framework), human behavioural flexibility, including in the vocal realm, allows vocal co-timing to play a role in more complex social behaviours. We can flexibly deploy rhythm to selectively exploit some of its fundamental principles. Rhythm allows sophisticated prediction and cooperation. Consider speech acts like “Jump on three: one…two…three.” Timing turn-taking in speech avoids noisy overlap of signaling, but also helps orchestrate attention to the content of speech [20]. Further, rhythm’s predictability may be further enhanced by its facilitation of representation. Copious evidence indicates that rhythmically structured speech (nursery rhymes, historical oral epics, etc.) is easier to remember [21–24]. This is likely due to the fundamentally rhythmic nature of cross-system neural coordination, almost certainly involved in complex cognitive representations [25]. There is likely evolutionary bootstrapping between the ability to perceive and produce complex rhythms.

To what extent is flexible and complex use of vocal rhythm dependent on adaptations beyond heavily conserved aspects of neural rhythm and predictability? Do volitional vocal control and social coordination co-evolve? To answer these questions, we need to compare flexible rhythmic behaviour in social contexts across related species with systematic variation in these traits. Despite the florid use of social vocal rhythm in humans, primates are not the most promising extended clade to answer these questions. This is because, despite the commonality of rhythmic vocalizations in primate species, there is very little vocal synchrony or coordination in the great apes beyond inhibition and vocal contagion [26–29]. This is despite their ability to coordinate other types of rhythmic behaviour (walking, facial expressions, etc. [30, 31]). Notably, gibbons may be an exception [32], and evidence for rhythmic flexibility is mounting in indris (e.g. [33, 34]). Primates may be limited in precise control of their vocal timing in part by a lack of volitional larynx and vocal tract control [28, 35], although here marmosets may be an exception [36]. Because humans seem to have accumulated a great many traits related to vocal timing and sociality, with limited distribution of these traits among the most closely related primates, the evolutionary history of vocal timing and sociality in primates is obscured.

How did humans, coming from a line of species with relatively limited vocal plasticity, evolve to express such high vocal plasticity across domains? There are theories that volitional breathing control in an early hominid was a preadaptation for later vocal plasticity and language [37]. In most mammals, vocal output is inextricably linked with the timing and control of breathing. Because breathing is obligate and automatic in most species, this also limits the extent to which vocal output can be volitionally and flexibly deployed. Did volitional control of breathing, and thus flexible timing of vocalization, serve social coordination functions prior to the evolutionary emergence of language?

While volitional vocal control, and thus flexible vocal coordination, is rare and limited in most mammals [38], there is one group of species that is distinguished by widespread control of breathing, flexible vocal behaviour, and social rhythmicity: the marine mammals. In this Perspective, we focus on the two best-studied clades in this group, the cetaceans and pinnipeds. Between them, they produce vocal rhythms that span the entire tempo range observed in animal vocal communication from 0.1 to more than 1000 sounds per second [39], have highly divergent vocal production flexibility up to mimicry in some species [40], and have a range of apparently rare vocal adaptations, including direct cortical connections to phonatory brainstem neurons in some species [41]. They also express a wide range of social structures, from nearly solitary to fission–fusion megapods and extended matrilineal kin relationships [42]. This allows us to examine potential co-variance between social structure and capability for, and usage of, vocal rhythms. Thanks to advances in genetics, the cetacean and pinniped phylogenies are well established [43, 44], and presence and absence of distinct traits related to social vocal rhythm will inform parsimonious evolutionary models.

The cetaceans are an intriguing group to examine due to empirically verified behavioural synchrony and vocal synchrony in affiliative contexts. Notably, male Indo-Pacific bottlenose dolphins (*Tursiops aduncus*) use synchrony as a social tool to express social bonds and facilitate cooperative social interactions [45, 46]. This includes the use of synchronous motor displays by pairs or trios of allied males when cooperating, and the use of vocal synchrony in which allied males appear to match the tempo and production of vocal signals as a bonding mechanism. While the neurobiology of toothed whale vocal motor control has not yet been studied, peripheral examination of their sound production mechanisms reveals a number of adaptations that promote vocal timing in the millisecond range [47]. In addition, there is evidence of evolutionary convergence of vocal plasticity in the nasal complex of toothed whales, allowing them to vocalize in different vocal registers: lower vibration rates for clicks and higher vibration rates for whistles [48]. Despite their evolutionarily novel sound production mechanism, toothed whale voiced sound production is in keeping with the myoelastic-aerodynamic theory developed to explain laryngeal sound production [49]. In addition to precise interval timing and frequency modulation, toothed whales produce vocal rhythms that span the entire range of vocal rates produced in the animal kingdom. The most rapid vocalizations come from harbour porpoises (*Phocoena phocoena*), which can click in isochronous trains at rates up to 1200 clicks per second [50]. On the other end of the spectrum, sperm whales (*Physeter macrocephalus*) have been shown to produce highly isochronous clicks with precise intervals as large as 10 s [51].

Cetacean calls also demonstrate rhythmic complexity beyond isochronicity. Notably, sperm whales produce heterochronous “codas” (stereotyped complex patterns of clicks with high timing accuracy), demonstrating an ability to produce, mimic, and alter rhythmic structure [52, 53]. Among the baleen whales, humpback whales (*Megaptera novaeangliae*) produce songs with heterochronous rhythmic characteristics, although the complexity and repeatability of their patterns has only just begun to be characterized [54, 55]. Because every cetacean must be able to organize its breathing for aquatic life, we posit that basal adaptations in their ancestors released constraints on breathing and thus call timing. Vocal plasticity may then have been further selected for by the importance of vocal communication in maintaining social contact in the low-light underwater environment. The variability in cetacean vocal plasticity and timing can be compared to the variability in social structure, ranging from the relatively asocial porpoises through the structured, multilevel societies in sperm whales (*Physeter macrocephalus*), short- and long-finned pilot whales (*Globicephala macrorhynchus* and *Globicephala melas*), and orcas (*Orcinus orca*) [56].

While there is less evidence of social synchrony in pinnipeds, seals and sea lions perform call and response mother–pup contact calls [57], and distinguish themselves compared to terrestrial mammals in terms of vocal flexibility [58] and rhythm [59–61], while expressing a broader range of social structures than the cetaceans. Three pinniped species from the sea lion (family Otariidae) and true seal (family Phocidae) lines have now been shown to have direct cortical connections to brainstem phonatory neurons, as humans and songbirds do [41]. The phocid pinnipeds have several other neural adaptations supporting vocal production flexibility. While the sea lions and fur seals have not been demonstrated to learn new vocalizations over their lifespan, they are capable of easily controlling the timing of their calls [62]. Some southern seal species (*monachinae* phocids) have shown evidence of developmental call learning [63]. Walruses (*Odobenus rosmarus*) show preliminary evidence of extensive vocal plasticity across multiple dimensions [62, 64], and two species of seals have been shown to produce vocal mimicry of frequency and formant content [65–68]. Although pinnipeds have not been shown to co-time their calls in the wild (aside from the aforementioned call and response in mother–pup contact calls), a number of pinniped species do produce rhythmic calls, at varying levels of complexity [69, 70]. In addition, in the gross-motor domain, a California sea lion (*Zalophus californianus*) is currently the non-human animal that has shown the most precise and consistent flexible synchronization of movement to rhythmic stimuli, now performing at a level as good or better than typical adult humans [60]. Pinnipeds also express a wide range of social organization [71, 72], allowing comparisons between vocal plasticity and timing and sociality. Some phocids are almost wholly solitary outside of the breeding season and have exceptionally brief nursing periods. Walruses and many of the otariids, in contrast, live in large, gregarious social groups, maintain some social bonds over time, even extra-familial, and have shown tantalizing evidence of social coordination of hunting [73, 74].

The marine mammals offer a unique opportunity to unravel the various mechanisms and social contexts of vocal rhythm across whole phylogenies. This in turn will allow modeling the evolution of the complex, interrelated aspects of social vocal rhythm in mammalian species where vocal capability is more patchily distributed. Below we discuss facilitation and constraint of vocal rhythm by peripheral sound production mechanisms (Section “Peripheral sound production mechanisms”) and the central nervous system (Section “Central mechanisms for rhythmic sound production”) in pinnipeds and cetaceans. We address current knowledge of the social co-timing in marine mammals (Section “Social functions of rhythm”). We then propose how current evidence suggests a virtuous cycle of evolutionary modeling and empirical exploration of social vocal synchrony in the marine mammals (section “Evolutionary, mathematical, and computational models of social rhythms”).

## Peripheral sound production mechanisms

Despite clear evidence of flexible vocal production timing in marine mammals, any serious catalogue of their vocal rhythm must begin with the peripheral physical mechanisms of sound production. These mechanisms can be the target of flexible central nervous system control, but their physical characteristics are limiters of that flexibility. No matter the skill of the player, a tuba cannot sound like an oboe. In addition to limitations, the physics of sound production may also present opportunities for rhythmic production that an animal can flexibly exploit. For example, the resonance properties of tissues may allow animals to produce steady output of discrete sounds without the need for neural pacemakers or volitional timing.

Toothed whales, baleen whales, and pinnipeds all produce sounds by driving pressurized air across vibrating tissues in their respiratory tract. Pinnipeds and baleen whales use vocal cords as in terrestrial mammals [75], whereas toothed whales have lost their vocal folds in the evolution of a unique de novo sound production mechanism in their nasal system [48, 76].

The main challenges of producing sounds underwater are related to dramatically diminishing air volumes with increasing hydrostatic pressure at depth. In the absence of new air to inhale, diving mammals are either limited to a single vocalization per submergence, or must utilize a mechanism to recycle air between vocalizations [75]. Some pinniped vocalizations, such as harbour seal (*Phoca vitulina*) roars, do indeed involve air exhalation to the water, limiting the seals to one vocalization per submergence. However, the rate and hence rhythm at which such dives can be undertaken may offer honest advertisement clues to diving and lung fitness of vocalizing males. Other pinnipeds species, such as bearded seals (*Erignathus barbatus*), can seemingly recycle air between vocalizations and hence undertake long song displays with rhythmic information while submerged, although the exact mechanisms for such recycling are not known.

Baleen whales have evolved unique modifications to their vocal cords and associated tissue that allows them to vocalize when air moves across them from both directions [77]. These adaptations allow for continuous sound production during breath-hold dives, but excitation of the vocal folds require high air volumes, limiting baleen whales to making sounds in the upper 100 m of the water column. There is compelling evidence for bi-phonations suggesting that some baleen whale species can excite two separate sources in the vocal tract simultaneously [78].

Toothed whales produce sound by forcing air across two pairs of phonic lips in the nasal complex that are excited in different tension-flow related vibration regimes to enable at least three different vocal registers of clicks, burst-pulses, and tonal sounds [48, 79]. Clicks are mainly produced by the right phonic lip pair, while burst-pulses and whistles are mainly produced by the left phonic lip pair [80, 81]. These dual sources allow for the production of individually controlled biphonations at different rates [81]. Biomechanical resonance may naturally allow for rhythmic sound production in the parts of the vocal repertoire of toothed whales that involve clicks and burst pulses. This includes stable echolocation clicks in most species [82], as well as modulated burst pulses in narwhals [83, 84] and porpoises [50]. The air that acts as a propellant for exciting sound-producing vibrations in the phonic lips is collected in vestibular air sacs just below the blow hole and, after a series of phonations, is recycled back down past the phonic lips for a new sound production cycle [85]. These recycling events occur at increasing rates with increasing depth, and because the production of tonal sounds involves higher airflows across the phonic lips than click production, deep diving toothed whales seem to face constraints on the depth at which tonal calls can be made [86]. The nasal sound production in toothed whales, powered by air pressurized anterior to the epiglottis, also uniquely allows them to (1) produce very loud sounds via high air pressures that would destroy the lung epithelium of terrestrial mammals that all drive vocalizations via air pressurized in the lungs, and (2) vocalize while ingesting prey. By contrast, pinnipeds and baleen whales face the same risk of suffocation as terrestrial mammals trying to swallow and vocalize at the same time.

Thus, pinnipeds, baleen whales and toothed whales all use air-propelled sound production and can recycle air. This evolutionary capability allows them to keep vocalizing with one lung-full of air independent of locomotion and breathing. This also means that when recycling air, there is a pause to vocalizations that ultimately limits the rate or duration of call epochs, and that long tonal sounds cannot be produced at great depths where air volumes are greatly diminished owing to Boyle’s law. Thus, these breath-hold divers are ultimately limited in their vocal displays by their dive durations and dive depths.

Feeding adaptations may also affect call timing, as exemplified in the pinnipeds: Seals and walruses, although not eared seals (sea lions and fur seals), utilize underwater suction feeding and underwater nursing [87–89], both of which are supported by supralaryngeal and apparently volitional control of mouth and tongue structures also involved in the upper vocal tract. Movements of the mouth and tongue in these species can allow fine-grained and temporally precise alteration of vocal output [58]. Walruses are particularly notable for the wide range of vocal types they produce, both in the wild and under stimulus control in captivity. These include clicks, grunts, barks, groans, and even whistles [62]. Male walrus “songs”, produced during the mating season, contain a number of rhythmic elements, including knocks and “bell” sounds that can be produced in stereotyped and repetitive sequences [90, 91].

Across the marine mammals, adaptations for breathing and feeding contribute both to the character and timing of calls in the second and millisecond range, and to the potential for volitional control of call onset and offset. This range of adaptations provides groundwork for considering what aspects of call timing are due to top-down, volitional control, and in what ways marine mammal calls are rhythmically flexible and available for recruitment in social contexts where timing matters.

## Central mechanisms for rhythmic sound production

Just as peripheral mechanisms for sound production facilitate and constrain rhythmicity via resonance and breathing cycles, central nervous system mechanisms may contribute to call timing via a range of interrelated processes. Thanks to opportunistic ex vivo histology and magnetic resonance imaging (MRI) tractography, we have more detailed knowledge than ever before of the structure and organization of toothed whale and pinniped brains, and we are beginning to gain similar insight into the baleen whale brain. Marine mammal brains can contribute to call timing and social use of vocal rhythm both through broadly conserved, low-level neurobiological processes and specific, systems-level neural adaptations to brain structure and organization.

All nervous systems produce temporal patterns due to their fundamental biological nature. There is a constrained set of “pacemakers” in the nervous system, which are sets of neurons that fire at set rates and can output those rates to effectors [92]. These pacemakers can have different biological drivers, but tend to involve regular cellular gating of ions at a set rate leading to predictable action potentials. Pacemakers can play a role in regular periodic behaviour such as breathing [93]. Groups of neurons with rhythmic functional properties can assemble into central pattern generators, which control rhythmic behaviour conditionally, such as with chewing or locomotion [94]. Importantly, peripheral resonance mechanics (soft assembly) of bodies and how they interact with their environments can also drive steady motor rates without pacemakers or central pattern generation [95]. Unsurprisingly, given their role in breathing regulation, pacemakers have also been implicated in regulating vocal output in some species [96], although there are limited data on marine mammals. Some rhythmic patterns in marine mammal vocalizations may be due to the function of such pacemakers. However, in the absence of invasive neurobiological recording, it is often difficult to disentangle their contribution from the mechanical resonance of peripheral effectors, discussed in the previous section.

Pacemaker neurons are distributed throughout the cortex in mammals, where they are believed to help regulate firing rates and coordination of complex intracortical networks. The fundamentally rhythmic nature of the brain, however, is not ascribed to sparse pacemakers. Rather, it has been shown that neurons, the foundational units of the nervous system, operate as loosely coupled oscillators. The “firing” rates of linked neurons influence each other, with ensembles of neurons entraining and synchronizing during sensation, motor behaviour, and cognition [97, 98]. This temporal coordination is ubiquitous in animal brains, found across phyla, and is so foundational to neural organization that correlation of firing patterns is considered a sufficient criterion for demonstrating “functional connectivity” between distributed neurons and brain regions [99]. The temporal patterns that co-oscillating neurons and brain regions create span from milliseconds to seconds, and are involved in every brain process and at every level of the nervous system, from homeostatic regulation through cognitive control [100]. These central mechanisms of neural timing presumably operate in all marine mammal brains, as they appear to be a chordate neurobiological universal.

Synchrony is more the rule than the exception in the brain. This has been particularly closely studied in sensorimotor coordination [101, 102]. The bidirectional causality of sensory and motor processing depends on precise timing and coordination across distributed systems; movement in response to sensation, sensation in response to movement, and sensory and motor predictions in response to sensory and motor inputs must all coordinate to drive environmentally oriented behaviour. The ability to maintain this precision is likely necessary for hunting, predator evasion, and any complex group behaviour.

While coordination of skeletal motor systems with sensory perception is likely ubiquitous in mammals, vocal motor systems may be a notable exception to this rule. In most species, vocal behaviour is driven by midbrain and brainstem nuclei that inhibit and trigger species typical calls in certain contexts [103–105]. This is likely primarily due to the fact that the vocal apparatus is shared with the primary mechanisms for handling breathing and swallowing, both of which are automatic behaviours in most vertebrate species studied [106, 107]. A byproduct of this evolutionary arrangement is that, unlike the rest of the skeletal motor system, the vocal apparatus of most mammals is not directly integrated with the cortico-basal ganglia and cortico-cerebellar motor learning loops, both of which are essential for motor plasticity in response to feedback and experience. In the typical terrestrial mammal bauplan, laryngeal and vocal tract control nuclei in the brainstem (such as nucleus ambiguus) send direct efferent connections to vocal effectors. These brainstem nuclei are controlled by top-down connections from midbrain regions, predominantly the periaqueductal grey (PAG) [103, 108]. The midbrain regions inhibit and release the vocal production nuclei depending on organismal state, largely dependent on affect and arousal levels. This midbrain-brainstem-effector hierarchy is largely automated and is not particularly plastic across development [109, 110]. This helps explain an apparent paradox—despite much greater general motor plasticity, and much more neural “real estate” devoted to learning, bigger brained phyla such as birds and mammals show less vocal synchrony and chorusing than do amphibians and insects [111]. “Lower” phyla do not have the same tight overlap of breathing and vocal output that the birds and mammals do, potentially freeing their vocal output for susceptibility to alternative influences such as the timing of signals from conspecifics.

In some mammals, there are top-down cortical connections with the PAG that can influence the PAG’s signaling to the brainstem phonatory nuclei. Most notably, anterior/rostral cingulate, which is involved in decision making and cognitive conflict resolution, can send strong efferent connections to the PAG that can inhibit or facilitate its activation [112]. Because the cingulate, part of the extended Papez circuit, is integrated with reward circuits and helps deconflict competing behavioural impulses [113], this is potential flexible leverage over onset/offset plasticity of stereotypic vocalizations, and thus, call timing. The extent to which this type of control via the PAG allows for flexible timing, and under what affective and arousal states, remains to be determined across species.

Compared to typical terrestrial mammals, the marine mammals have a potential cross-clade advantage for flexible timing of calls. Marine mammals may have experienced adaptive pressure to alter neural control of breathing to support dive behaviour and vocalization at and below the surface. Although these adaptations have not been systematically assessed in all marine mammals, there is emerging evidence for enhanced volitional breathing control in multiple species. Grey seals (*Halichoerus grypus*) do not rely on carbon dioxide levels in the blood, as terrestrial mammals do, to drive obligate and volitional breathing, and instead are capable of planning dive duration based on current O_2_ blood concentrations [114, 115]. Under general anaesthesia, cetaceans and seals have been shown to engage in extended apneas, and, in certain cases, will simply not breathe even to the point of potential death [116]. Even the fur seals and sea lions, among the most terrestrially competent marine mammals, can engage in apneas when awake [117]. Note that this suggests more extreme volitional respiration adaptations than are found in humans, who, despite our high degree of flexible volitional breath control, lapse into automatic regular, rhythmic breathing when not attending to respiration or when unconscious. While more work is required to clarify the physiological and neurobiological mechanisms of breathing control in different marine mammal species, it may be that aspects of their breathing are more volitional or non-obligate than in most terrestrial mammals. This might help explain the unusual sleep neurobiology of marine mammals, some of which rely on unihemispheric sleep, only achieving slow wave sleep in one brain hemisphere at a time [118], thus potentially letting the other hemisphere contribute to volitional breathing behaviour. Together, these findings support the possibility of enhanced cortical volitional control of breathing behaviour in these species.

One fairly straightforward evolutionary explanation for these breath control behaviours is that, when diving, an involuntary inhalation would likely be fatal. Perhaps, in marine mammals, inhibition on breathing motor systems must be volitionally released, similar to how many perching birds have to volitionally open their claws to let go of a branch [119]. If volitional movement of the breathing apparatus has increased in marine mammals, this could in turn release constraints on volitional control of vocal behaviour as well. New data in pinnipeds supports just such an interpretation. Sea lions and seals, but not a terrestrial outgroup carnivore species, have direct, robust, bilateral connections between vocal motor cortex and the nucleus ambiguus, one of the nuclei controlling laryngeal muscles [41]. Because the ambiguus, a vagus nerve nucleus, is involved in breathing and swallowing, as well as vocalization, Cook et al. (2026) suggest that this pathway evolved to support volitional control of breathing (and potentially swallowing behaviour) for amphibious life. Interestingly, the seals also showed increased connectivity in premotor cortical circuits related to vocal production flexibility in birds and humans, while the sea lions did not. Sea lions have been demonstrated to easily place species typical calls under stimulus control [120], but, unlike the phocids, have not been shown to alter the frequency and filter aspects of their species typical calls [121]. These brain circuits have not been assessed in cetaceans, but are a promising research target. What *has* been shown in a range of cetacean species is developmental call learning and even vocal mimicry, suggestive of volitional control of the vocal apparatus [40]. With greater degrees of freedom on vocal motor control, marine mammals may have been well endowed for adaptations supporting vocal coordination. As discussed in the following section, there is some strong emerging evidence of just that.

Importantly, even if a species has increased capability for volitional control of vocal behaviour, including call timing, there will almost certainly be aspects of call timing that are reliant on physiological state, peripheral effector structure, and central pattern generation. For example, despite extensive volitional control of vocal behaviour, humans still produce largely automatic vocal outputs in social contexts, some of which can be rhythmic. Consider laughter and crying. Both of these tend to be automatic once triggered, and also frequently have isochronous timing [122, 123]. However, although these call types are not fully volitional, we can still flexibly organize our social behaviour around them. We may flexibly and volitionally seek circumstances in which we are likely to laugh, and may also flexibly and volitionally seek to make others laugh.

**Fig. 1:**
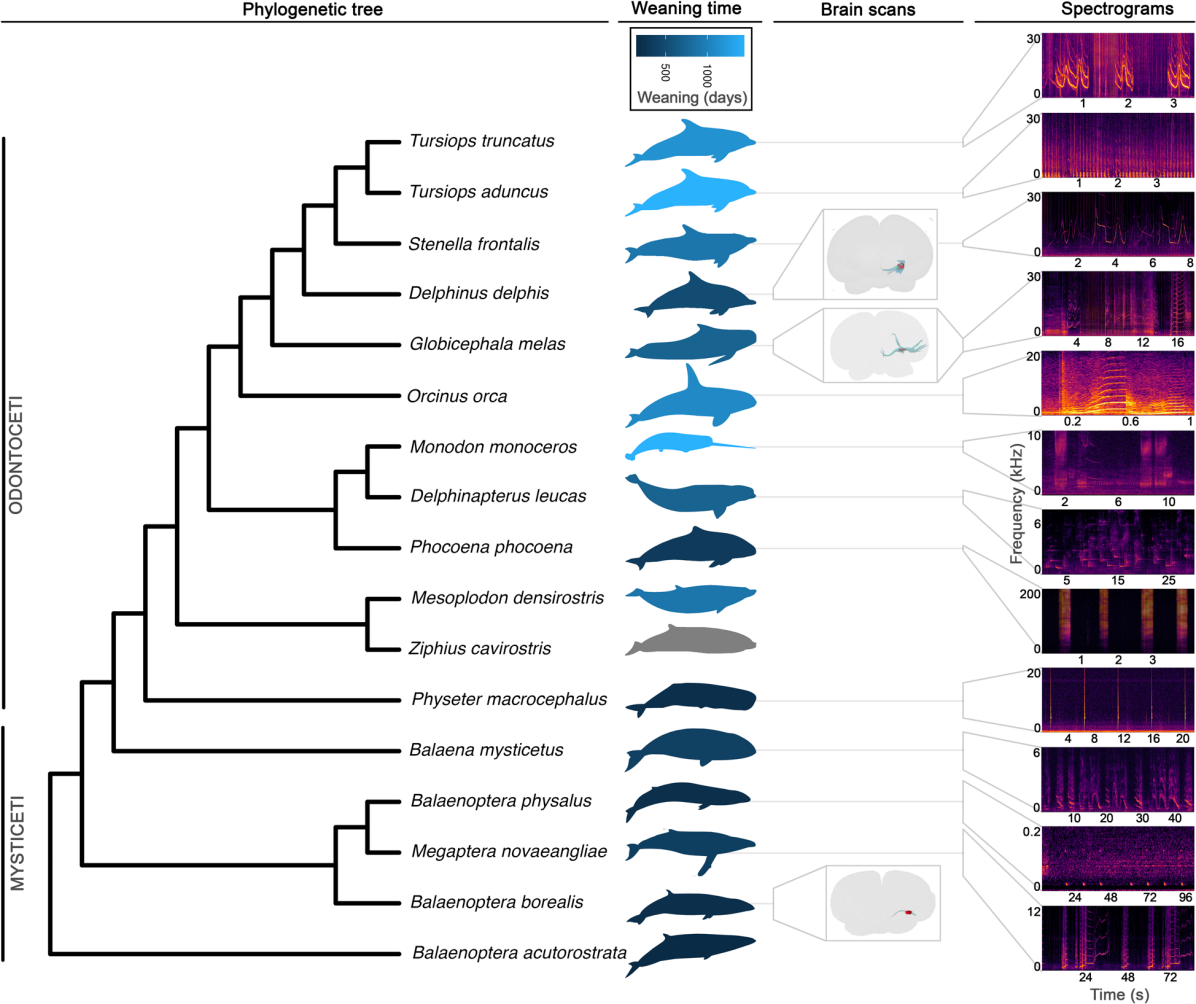
Social and vocal complexity in cetacean species. Cetacean phylogeny is presented along with aspects of their biology potentially related to the complexity of their social lives and communication signals. Age at weaning (defined here as mean age at which young are no longer dependent on nursing for survival), one proxy for social complexity, is colour-coded with darker blue for animals weaned earlier and lighter blue for animals weaned later. Amygdala connectivity—related to socioemotional processing in terrestrial mammals—is shown for three species, generated via probabilistic tractography from post-mortem brains scanned opportunistically. Wild-recorded vocal communication signals are shown in spectrograms with non-matched timescales. Thanks to technological advances in data collection and processing, obtaining and integrating these multimodal data cross-clade is newly possible. Similar analyses can be conducted across pinniped species. Brain scans and spectrograms are drawn from works in progress. Weaning times were collated from the existing literature (see Supplementary Table 1)

Assuming enhanced volitional control of vocal behaviour in marine mammals, there is still substantial work to be done to determine how marine mammals perceive and produce rhythmic vocalizations. In humans, there is strong evidence that different brain circuits serve different aspects of volitional timing. Broadly speaking, the cerebellum handles precise event and interval timing [124, 125], while the basal ganglia are essential to recognizing and extracting relative timing from complex sequences [126, 127]. In humans and non-human primates, premotor and supplementary motor regions also seem to play a role, potentially by creating a motor simulation of rhythmic patterns that can then drive entrained motor output [126, 128, 129]. Interestingly, these regions play similar and complementary roles in both perception and production of behavioural rhythms. Each of these regions could be assessed in marine mammals. While invasive work is off the table for these species, in vivo neuroimaging can be implemented with pinnipeds [130, 131], and post-mortem assessments of neural connectivity have been successfully used with pinnipeds and cetaceans [132–136] (Fig. 1).

The cerebellum has been singled out in the toothed whales; it is apparently hypertrophic, taking up a much larger proportion of total brain volume than in most terrestrial mammals [137, 138]. This has been suggested to play a role in echolocation [139]. The cerebellum operates in part as a Bayesian sensorimotor prediction system, using the current organism state to make a feed-forward prediction of motor outcome, and then updating based on action outcome [140, 141]. This might be particularly important for very fast processes requiring precise and dynamic sensorimotor coordination. In echolocation, outgoing clicks can return an echo faster than the time required to hear and process the echo and alter vocal behaviour before the next click [142]. Therefore, one might think of echolocation as a dynamic tuning process whereby outgoing motor behaviour is based on rapid prediction of likely outcomes, taking into account current state and prior experience. Such processes may be heavily cerebellum dependent. In support of this, new data from post-mortem diffusion tractography indicates much denser cerebellar-auditory integration in toothed (echolocating) than baleen (non-echolocating) whales [136]. Additionally, among the toothed whales, species producing faster echolocation click trains tend to have proportionately bigger cerebella [139, 143]. Importantly, the production of fast clicks is likely dependent on peripheral effector size and oscillation properties, not central nervous system activity. It is the prediction and perception of rapidly returning clicks and the integration of these processes with tuning continuing outgoing click speed that might be particularly computationally demanding with high duty cycles. There are fewer data on pinniped cerebellum, but there is some evidence of altered basal ganglia organization in carnivores, including marine carnivores [144, 145]. The caudate is much expanded in comparison to the putamen, a pattern that privileges goal-oriented cognition and sensorimotor transformation as opposed to fine-grained motor manipulation. This might aid in establishing and maintaining representations of temporal structure relevant to future motor behaviour.

Behavioural assessments of vocal rhythm in marine mammals, and rhythmic behaviour and perception in general, will also be informative and drive predictions and hypotheses about neural organization. Once brain regions and circuits related to timing have been quantified and compared between species with established social vocal behaviour, we can begin to examine the relationship between brain regions involved in timing and brain regions involved in social cognition. There are essentially no data on the “social brain” in marine mammals; future work may follow the path laid out in extensive work on primates [146, 147]. The highest and most complex expression of social vocal timing likely emerges from intricate interaction between heavily adapted timing and social brain regions.

## Social functions of rhythm

Having reviewed peripheral and central mechanisms contributing to temporal structure of vocal behaviour, we now turn to consideration of potential social functions of rhythmic vocalization. In the marine mammals, best current data are in the cetaceans. Rhythm is ubiquitous in the social lives of whales and dolphins, manifesting in sexually selected song displays, social bonding and cooperation, coalition quality, infant care, and other types of social interaction. Such rhythmic behaviours occur in both the vocal and motor domains, and the larger interspecific diversity in socio-ecology among marine mammal species makes them an ideal group of animals with which to investigate the evolution of social rhythm [39].

Synchrony, which involves repeated events that occur at the same time, is a form of social rhythm frequently observed among cetacean species. At the simplest level, synchronous surfacings or synchronous breathing, where two or more animals break the water’s surface in unison to breathe, are widespread in whales, dolphins, and porpoises [148]. Synchronous surfacing is a predominant behaviour in bottlenose (*Tursiops* spp.) and Commerson’s dolphin (*Cephalorhynchus commersonii*) mother-calf pairs within the first month of life [149, 150] and is believed to be an important mechanism for facilitating affiliation and learning [151]. Similarly, mother-calf humpback whale pairs synchronize their surfacings and general dive behaviour [152–154]. For both dolphins and baleen whales, it has been suggested that close-proximity mother-calf synchrony may provide hydrodynamic benefits [155] and allow the mother to deliver continuous maternal care and protect the calf against predation [152]. Synchronous breathing is not limited to mother-calf pairs. In bottlenose dolphins, it is an important component of affiliative behaviour between allied males [45], and same-sex pairs of similar age will frequently engage in synchronous breathing [148]. Further, male-male synchronous breathing has been shown to be a strong indicator of association strength in Risso’s dolphins (*Grampus griseus*) [156] and proposed as a useful tool for studying pilot whale sociality [157]. Synchronous breathing is significantly more frequent between bottlenose dolphin pairs when exposed to anthropogenic noise, which may be an anti-predator response or action to promote social cohesion when acoustic communication is compromised [158]. Other species also engage in extended bouts of synchronous diving; male-male pairs of Cuvier’s beaked whales (*Ziphius cavirostris*) showed extended periods of synchrony in diving behaviour, although the function of this male-male synchrony remains unclear [159]. Further, both Blainville’s (*Mesoplodon densirostris*) and Cuvier’s beaked whales engage in extreme foraging synchrony, where deep dives are highly coordinated even though they hunt for prey individually, which appears to be an anti-predator strategy [160]. In Indo-Pacific bottlenose dolphins, behavioural synchrony is used as a social tool to express social bonds and promote social unity among allied males [161]. Such behavioural synchrony takes the form of synchronous surfacings and elaborate synchronous motor displays and aerial leaps [45, 161], as well as the synchronous production of isochronous vocalizations that largely occur when males are working together to herd single oestrus females [162, 163]. Such a prevalent use of cooperative synchrony by allied male dolphins supports the notion that synchronous behaviour evolved as a coalition signaling system in dolphin alliances [164].

Synchrony also plays an important role in cooperative foraging for many species. Both bottlenose dolphins and spinner dolphins (*Stenella longirostris*) will swim synchronously, line abreast before forming a circle to facilitate prey capture [165, 166]. Bottlenose dolphins in South Carolina will simultaneously swim side-by-side towards the shore and then surge out of the water in synchrony to capture small fish stranded by the surge wave [167–169]. Another famous example of behavioural synchrony in the context of cooperative foraging can be found in pack ice killer whales, where group members will synchronously charge underwater side-by-side towards an ice floe, lifting their tails and diving under the ice, creating a wave that breaks over the floe and typically washes seals into the water [170]. All these examples demonstrate how toothed whales have co-opted rhythmic behaviour (in the form of behavioural synchrony) to support their complex cooperative endeavours.

Rhythmic abilities are also strikingly apparent in the vocal domain of cetacean species, with isochronous rhythm being particularly prevalent [39]. Bottlenose dolphins produce individual identity signals, termed “signature whistles”, where the time intervals between repeated elements within a whistle are often isochronous [39]. Further, while signature whistles are produced in sequences with varying inter-whistles intervals [171], there appears to be an optimal time interval (≤ 1 s) within which individual dolphins should exchange whistles to elicit a vocal response [172, 173], supporting the idea that whistle production is under volitional control. Baleen whales are known for their production of isochronous and heterochronous song, which play a role in mate attraction and courtship (reviewed in [39]). The poster child for rhythmic song is the humpback whale [174], which produces hierarchically structured song that is shared between individuals and populations via social learning [175], creating rhythmic song patterns that are the foundation of one of the most famous examples of cultural transmission in the animal kingdom [176]. Isochronous vocal patterns also occur in toothed whales when socializing (long-finned pilot whale (*Globicephala melas*) [177]; sperm whales [178]; bottlenose dolphins [163]) and during aggressive interactions (bottlenose dolphins and Atlantic spotted dolphins (*Stenella frontalis*) [179]). Indeed, the rhythmic production of coda vocalizations in sperm whales appear to function as symbolic markers of cultural identity [180], revealing the role of rhythm in cetacean vocal cultures is perhaps not limited to song.

In pinniped species, there are limited data on synchronous behaviour and vocal rhythms. Notably, by almost any measure, pinnipeds are less socially complex than most cetaceans, providing a meaningful comparison clade to the cetaceans, where adaptations related to vocal control may be similar, but social pressures quite different. Odobenids and otariids do maintain long-term familiarity with conspecifics and have fairly extended maternal care [71, 89, 181–183]. Preliminary work shows fairly extensive play behaviour in some pinnipeds [184, 185]. Emerging data hint at complex social foraging in the sea lions, but more data are needed [73, 74, 186, 187]. Most seal species tend to be fairly asocial outside of the breeding season and have extremely brief maternal care periods [188].

Phocid seals produce some of the most complex calls among pinnipeds, but these are predominantly during breeding displays, and do not tend to be produced extensively in other social contexts [189]. Northern elephant seal (*Mirounga angustirostris*) males produce rhythmic vocalizations in breeding displays, and there is evidence that other males recognize these calls by their rhythmic structure [190]. The sea lions and fur seals, despite their limited vocal repertoire, do vocalize regularly in social contexts, most typically the iconic “bark”, which is produced both underwater and at haul outs [191], serving primarily as an aggressive signal during territorial disputes [192, 193]. Barks can be produced at a range of rates and amplitudes, but are typically quite isochronous, falling between 1 and 4 Hz repetition rates [69]. There is no evidence of bark synchronizing. Mothers and pups engage in call and response vocalization, but at very slow rates, typically one to four signals per minute in most species [57]. Importantly for pinnipeds, the bulk of maternal care is delivered on land, a characteristic that may partially account for their reduced social vocal synchrony in comparison to the cetaceans. The presence of vocal plasticity and the capability for isochronous vocal signaling make the apparent absence of social vocal synchrony in the pinnipeds a striking contrast to what has been observed in some cetaceans.

The presence and absence of different traits related to sociality and vocal rhythms can be assessed across marine mammals phylogenetically. A detailed understanding of the evolution of rhythm goes hand in hand with precisely pinpointing which biological function(s) a rhythmic behaviour serves in a species. Understanding the function means understanding the behaviour’s adaptive value and the possible evolutionary scenarios under which the behaviour can emerge. With few exceptions, it is impossible to directly observe which distal/evolutionary biological function a trait serves in a species. One can only observe the behaviour in different contexts in nature and under different experimental conditions, and infer the function based on this. However, evolutionary models offer us a way to explore the combinations of traits and mechanisms that might plausibly lead to the emergence of complex social vocal behaviour in species that are under different socio-ecological pressures.

## Evolutionary, mathematical, and computational models of social rhythms

Within animal rhythm research, vocal rhythms are a promising subject for evolutionary game theory and agent-based models. First, vocal instances of rhythm are in several cases known or hypothesized to be a form of communication [7, 194]. Given the inherent interactive nature of communication, one may ask how rhythmic vocalizations from single individuals interact within a larger population. The interaction between multiple simple mechanisms can often result in complex emergent behaviour, such as group rhythmicity, synchrony, and antiphonal calling. Second, vocal rhythms propagate in a shared medium. Individuals do not have a “private”, direct channel to communicate, and overlap and avoidance become crucial for successful communication. This effect emerges, either instantaneously or over an evolutionary scale, from individuals’ interactive behaviour. A modeling approach can simulate the interaction between known or hypothesized interactive behaviours, predict a group rhythmic outcome, and match this resulting outcome to empirical observations.

**Fig. 2:**
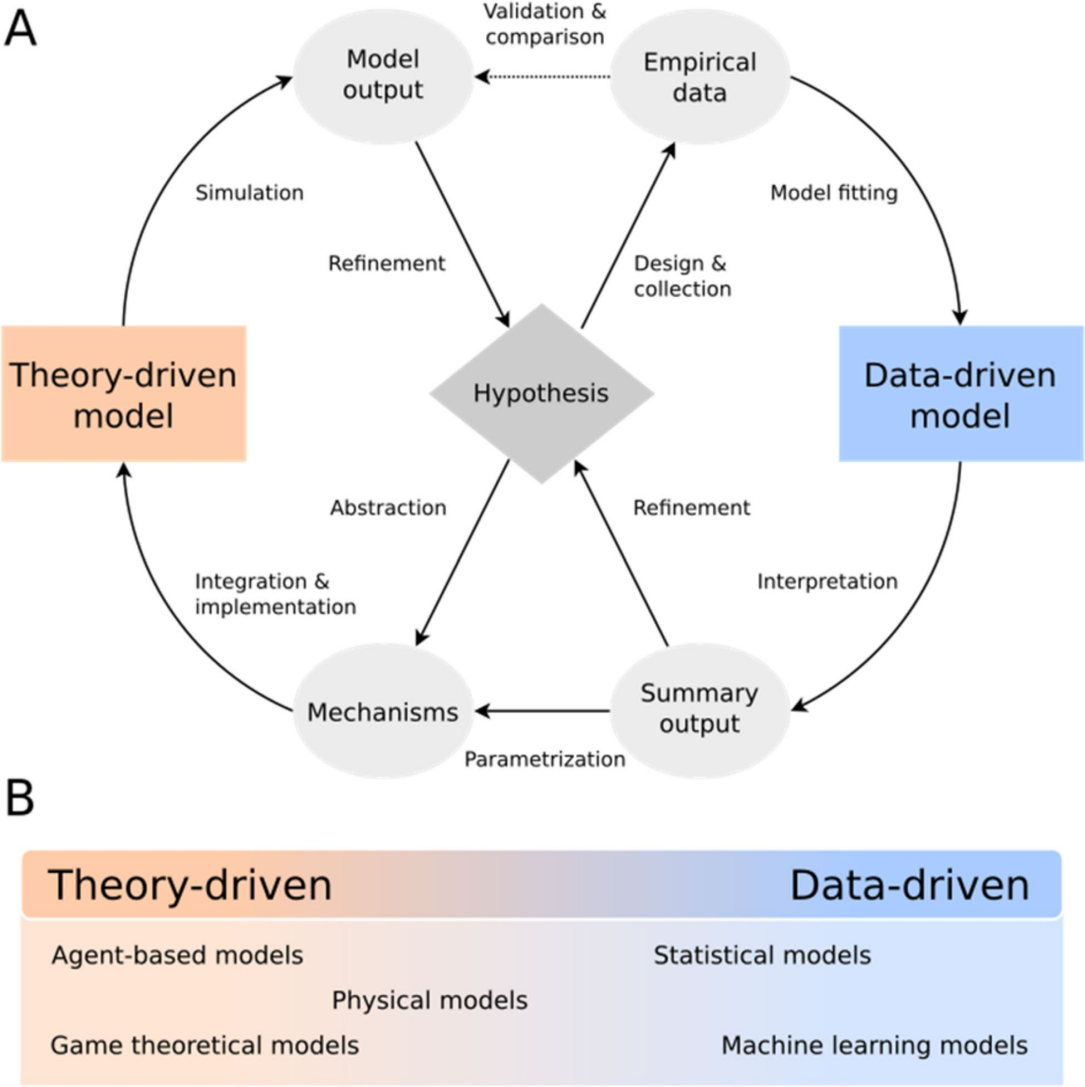
**A** The application of a computational model within the scientific process can be broadly classified into two categories: data-driven and theory-driven models. Typically, statistical models and machine learning models are applied in a data-driven manner: Starting from a hypothesis, one designs observations or experiments and collects raw empirical data. The data-driven models are fitted to capture the data in a simplified, structured manner, and can be interpreted to obtain a summary of the observational measurements or experimental effects. Complementarily, agent-based and game theoretical models are most often applied in a starkly different manner: Focusing on a phenomenon’s hypothesized mechanisms, one distills a number of simple but carefully specified mechanisms. Together, these mechanisms are combined into a single model integrating the proposed mechanisms, and implemented computationally. The theory-driven model simulations are both (1) potentially informed by the results of previous empirical studies and (2) validated through comparison to real-world data, confirming or rejecting the hypothesized mechanisms. Based on the results of either type of model, the current hypothesis can be updated, refined, or even rejected, and the whole process is repeated. **B** Modeling approaches lie on a continuum, characterizing how theory-driven (left side) vs. data-driven (right side) they are. On the one hand, agent-based models and game theoretical models are most often applied in a theory-driven way, resulting in models with limited input from empirical data. On the other hand, classical statistical models and machine learning models are often much more data-driven, explaining the structure and effects in empirical data. In this specific depiction, for instance, game theoretical models are equally theory driven as agent-based models; however, either of those is less data-driven than statistical models

Traditionally, computational modeling has provided crucial tools to synthesize the available data and hypotheses. Commonly used statistical modeling techniques, from *t*-tests to GLMMs, have long been an essential tool to summarize empirical data and test the significance of observed effects. However, the research questions and data presented above also lend themselves to investigation through evolutionary modeling (theory-driven models; see Fig. 2). Evolutionary modeling does not gradually disentangle general principles from empirical data (right side of Fig. 2); instead, it simulates varying evolutionary scenarios based on hypothesized mechanisms (left side of Fig. 2). In this complementary approach, the simulations’ results are then compared against natural observations to assess whether the hypothesized mechanisms can provide a feasible explanation.

Both types of computational modeling could be used to study vocal rhythm in marine mammals. As an example, take the vocal synchrony of dolphin “pop” vocalizations and the complex social structure of dolphin alliances. Previous research has documented male dolphins producing narrowband, pulsed “pop” vocalizations in repetitive trains [195–197]. These are produced only by males, almost exclusively when herding a female, and act as an agonistic “come-hither” signal, inducing the female to stay close to the popping male. Crucially, males will coordinate—or synchronize—pop production when consorting a female together, while also synchronizing their physical behaviour [45, 163]. Studies have shown that males perform physical synchronous displays for longer after engaging in affiliative contact behaviour and that those males with weaker social bonds engage in more precise motor synchrony with each other [161, 198]. Further, males with stronger social bonds engage in vocal synchrony at higher rates [46]. This supports a hypothesis of vocal synchrony as a social bonding mechanism, where male dolphins use synchrony to both maintain and strengthen their social bonds. So, how does social structure affect, and in turn how is it affected by, individually rhythmic and pairwise synchronized vocal displays? Below we sketch how this question can be addressed using two complementary approaches. First, we show a data-driven modeling approach, and then a theory-driven one.

A recent empirical study [46] provides an excellent example of a data-driven modeling approach (right side of Fig. 2). Chereskin et al. (2024) went to the field and collected both observational data and sound recordings. Their goal was to measure and model the social network of a population of dolphins, quantify the temporal properties of pop trains they produced, and assess the contexts in which they employed synchronous pop trains. The results of their analyses were then fed into several mixed-effects models. The resulting fitted model directly captures an idealized relationship between social factors and vocal synchrony, allowing for a clear, direct interpretation of the results: social variables numerically predict the degree of pop synchrony.

A parallel theory-driven model would constitute a complementary, though radically different, approach (left side of Fig. 2). Rather than inferring social networks from the observational data and measuring vocal synchrony in audio recordings, one could develop from scratch a simple yet explicit model of synchronization. For example, one could model each individual dolphin as a so-called oscillator, namely a mechanism repetitively producing pops at an intrinsic rate. The interactions within a social network of dolphins could be captured by the so-called Kuramoto model, a model of synchronization between two or more coupled oscillators [199, 200]. In its general formulation, synchronization in such a Kuramoto model is dependent on the coupling strengths between the individual oscillators. In our particular case, one could model dolphins as simple oscillators and the strength of social bonds between pairs as the coupling strength between these oscillators. In other words, one can encode possible social networks and simulate their interaction in a simple agent-based model. Such a model would allow modelers to systematically quantify the effect of the social structure on the agents’ rhythmic synchronization, and to compare the simulated levels of synchrony to real-world observations. Note that such an agent-based model can be gradually extended with other mechanisms; for example, one could investigate the effect of a complex feedback loop as social networks get updated based on the levels of synchrony in a population.

For both types of model, the predictions should be further tested empirically with field-based manipulations (e.g. playback experiments) to determine the function of vocal synchrony. The results of such a study can in turn validate or reject the proposed models and feed back into the improvements of the models and our overall understanding of the phenomenon.

As these concrete examples demonstrate, there is a potential advantage of combining these two different types of modeling. In general, the usefulness of data-driven models to inform and parameterize a new theory-driven model can be easily understood, but the combination of both types of models can in turn inform data collection and experimental design in at least three ways: (1) computational simulations are a relatively fast and inexpensive way of testing whether a set of mechanisms *could* give rise to an observed behaviour and (2) the exploration of different parameters can provide a focus for later data collection. Additionally, (3) theory-driven models force us to make existing hypotheses explicit in such a way that they can be implemented in a computational model. In the case of social rhythm and synchronization in marine mammals, computational and evolutionary models provide a particularly promising tool to capture complex social interactions and investigate the emergence of rhythmic behaviours. As we gather more data on the characteristics of social rhythmic behaviour in marine mammals, and as we develop new hypotheses on the evolutionary function, evolutionary game theory and agent-based models will verify or refute the proposed evolutionary trajectories and direct new research avenues with respect to empirical data.

## Conclusions

Multiple physiological mechanisms, both peripheral and central, can contribute to production of rhythmic vocalizations. These mechanisms are densely clustered and unevenly distributed across marine mammal species, allowing for a wide range of obligate and volitional temporally structured vocal behaviour. As we have argued here, this patchwork of characteristics, along with the wide range of social contexts in which cetaceans and pinniped rhythmic vocalizations have been recorded and characterized, makes the marine mammals an optimal set of model clades for disentangling the evolutionary course of social vocal rhythms. This in turn may provide novel and testable theories regarding the relationships between social affiliation and behavioural co-timing in a wide range of species.

To create plausible phylogenetic models of sociality and rhythmic vocalization across marine mammals, we need to record social vocal behaviour in the wild across cetacean and pinniped species. Much of this work has now been done in cetaceans, with large-scale acoustic recording arrays adding new data every day. Work on pinniped vocalization, particularly underwater, has lagged behind. We will also need to record behaviour during and around social vocalization—new work with drones and long-term spatial tagging are contributing to these efforts. Again, certain cetacean populations have been better studied than the pinnipeds, although data are most complete on a small subset of cetacean species. As we continue to determine which species do and do not engage in rhythmic social vocalizations, and in which contexts, we are better able to determine which biological mechanisms contribute to these complex behaviours. Of particular interest are the contribution of peripheral physiological systems (e.g. resonant vocal structures, breathing and air recycling systems) and central nervous regions and networks (e.g. subcortical timing centres and cortical systems for volitional control of vocal behaviour). These new data can feed into theoretical models that will then produce more testable hypotheses and provide direction regarding the most efficient and meaningful observations and experiments to test and elaborate evolutionary models. Integrated physiological and social ecology data will help us determine which traits predict social coordination in marine mammals, and possibly in other clades as well.

While we have focused here on peripheral and central mechanisms for rhythm and their relation to sociality, other intersecting domains of biology and ecology also bear on questions of the evolution of rhythmic sociality. Once physiological and neurological models of rhythm production are established phylogenetically, the genetic and molecular underpinnings of those traits should be explored. Productive cross-species genetic comparisons illuminating the evolution of vocal learning serve as a model [201, 202]. The role of environment and ecology may also be relevant. Signal repetition, relevant to rhythmicity, has been shown to correlate with background noise in songbirds [203, 204], and ambient noise can influence sound production strategy in humpback whales [205]. We welcome further integrative work on social rhythm in marine mammals beyond what we have proposed here.

This work is highly interdisciplinary, requiring acousticians, field biologists, evolutionary biologists, physiologists, behavioural ecologists, neuroscientists, and modelers, at a bare minimum. Scientific support and infrastructure for this type of high-level interdisciplinary science can allow for highly productive science where the emergent sum is not just additive from the joint expertise of scientists from different fields, but very likely multiplicative. In addition, we suggest that collaborations such as we propose here can also be carried out in other fields of biology and ecology, where dense local patchworks of field-specific information can be productively connected into highly informative mosaics.

## Supplementary Information

Supplementary Material 1.

## Data Availability

No datasets were generated or analysed during the current study.

## References

[1] Warner R. Rhythm in social interaction. In: McGrath JE, editor. The social psychology of time: new perspectives. Newbury Park, CA: Sage; 1988. p. 63–88.

[2] Bowling DL, Herbst CT, Fitch WT. Social origins of rhythm? Synchrony and temporal regularity in human vocalization. PLoS ONE. 2013;8(11):80402. 10.1371/journal.pone.0080402 PMC3843660 24312214

[3] Trainor LJ, Cirelli L. Rhythm and interpersonal synchrony in early social development. Ann N Y Acad Sci. 2015;1337(1):45–52. 25773616 10.1111/nyas.12649

[4] Launay J, Tarr B, Dunbar RI. Synchrony as an adaptive mechanism for large‐scale human social bonding. Ethology. 2016;122(10):779–89.

[5] Ravignani A, Honing H, Kotz SA. The evolution of rhythm cognition: timing in music and speech. Front Hum Neurosci. 2017;11:303. 28659775 10.3389/fnhum.2017.00303 PMC5468413

[6] Levinson SC. Turn-taking in human communication– origins and implications for language processing. Trends Cogn Sci. 2016;20(1):6–14. 26651245 10.1016/j.tics.2015.10.010

[7] Ravignani A, Bowling DL, Fitch WT. Chorusing, synchrony, and the evolutionary functions of rhythm. Front Psychol. 2014;5:1118. 25346705 10.3389/fpsyg.2014.01118 PMC4193405

[8] Hove MJ, Risen JL. It’s all in the timing: interpersonal synchrony increases affiliation. Soc Cogn. 2009;27(6):949–60.

[9] Greenfield MD. Synchronous and alternating choruses in insects and anurans: common mechanisms and diverse functions. Am Zool. 1994;34(6):605–15.

[10] Fried I, Haggard P, He BJ, Schurger A. Volition and action in the human brain: processes, pathologies, and reasons. J Neurosci. 2017;37(45):10842–7. 29118213 10.1523/JNEUROSCI.2584-17.2017 PMC5678016

[11] Hartbauer M, Römer H. Rhythm generation and rhythm perception in insects: the evolution of synchronous choruses. Front Neurosci. 2016;10:223. 27303257 10.3389/fnins.2016.00223 PMC4885851

[12] Greenfield MD, Marin-Cudraz T, Party V. Evolution of synchronies in insect choruses. Biol J Linn Soc Lond. 2017;122(3):487–504.

[13] Kelley DB. Vocal communication in frogs. Curr Opin Neurobiol. 2004;14(6):751–7. 15582379 10.1016/j.conb.2004.10.015

[14] Greenfield MD, Merker B. Coordinated rhythms in animal species, including humans: entrainment from bushcricket chorusing to the philharmonic orchestra. Neurosci Biobehav Rev. 2023;153:105382. 37673282 10.1016/j.neubiorev.2023.105382

[15] Ravignani A, Norton P. Measuring rhythmic complexity: a primer to quantify and compare temporal structure in speech, movement, and animal vocalizations. J Lang Evol. 2017;2(1):4–19.

[16] Jürgens U. Neural pathways underlying vocal control. Neurosci Biobehav Rev. 2002;26(2):235–58. 11856561 10.1016/s0149-7634(01)00068-9

[17] Scott SK. The neural control of volitional vocal production— from speech to identity, from social meaning to song. Philos Trans Soc Lond B Biol Sci. 1841;2022(377):20200395. 10.1098/rstb.2020.0395 PMC8591378 34775825

[18] Janik VM, Slater PJ. The different roles of social learning in vocal communication. Anim Behav. 2000;60(1):1–11. 10924198 10.1006/anbe.2000.1410

[19] Hoehl S, Fairhurst M, Schirmer A. Interactional synchrony: signals, mechanisms and benefits. Soc Cogn Affect Neurosci. 2021;16(1–2):5–18. 32128587 10.1093/scan/nsaa024 PMC7812629

[20] Thórisson KR. Natural turn-taking needs no manual: computational theory and model, from perception to action. In: Multimodality in language and speech systems. Dordrecht: Springer Netherlands; 2002. p. 173–207.

[21] Adams HF. A note on the effect of rhythm on memory. Psychol Rev. 1915;22(4):289.

[22] Brower C. Memory and the perception of rhythm. Music Theory Spectr. 1993;15(1):19–35.

[23] Thaut MH, Peterson DA, McIntosh GC. Temporal entrainment of cognitive functions: musical mnemonics induce brain plasticity and oscillatory synchrony in neural networks underlying memory. Ann N Y Acad Sci. 2005;1060(1):243–54. 16597771 10.1196/annals.1360.017

[24] Hoque E. Memorization: a proven method of learning. Int J Appl Res. 2018;22(3):142–50.

[25] Park HJ, Friston K. Structural and functional brain networks: from connections to cognition. Science. 2013;342(6158):1238411. 24179229 10.1126/science.1238411

[26] Hammerschmidt K, Fischer J. Constraints in primate vocal production. In: Oller DK, Griebel U, editors. Evolution of communicative flexibility: complexity, creativity, and adaptability in human and animal communication. The MIT Press; 2008. p. 93–119.

[27] Ghazanfar AA. Multisensory vocal communication in primates and the evolution of rhythmic speech. Behav Ecol Sociobiol. 2013;67(9):1441–8. 10.1007/s00265-013-1491-z PMC3821777 24222931

[28] Ackermann H, Hage SR, Ziegler W. Brain mechanisms of acoustic communication in humans and nonhuman primates: an evolutionary perspective. Behav Brain Sci. 2014;37(6):529–46. 24827156 10.1017/S0140525X13003099

[29] Ghazanfar AA, Liao DA, Takahashi DY. Volition and learning in primate vocal behaviour. Anim Behav. 2019;151:239–47.

[30] Ghazanfar AA, Takahashi DY. Facial expressions and the evolution of the speech rhythm. J Cogn Neurosci. 2014;26(6):1196–207. 24456390 10.1162/jocn_a_00575 PMC4441033

[31] Lameira AR, Eerola T, Ravignani A. Coupled whole-body rhythmic entrainment between two chimpanzees. Sci Rep. 2019;9(1):18914. 31831862 10.1038/s41598-019-55360-y PMC6908706

[32] Richman B. Rhythm and melody in gelada vocal exchanges. Primates. 1987;28(2):199–223.

[33] Gamba M, Torti V, Estienne V, Randrianarison RM, Valente D, Rovara P, et al. The indris have got rhythm! Timing and pitch variation of a primate song examined between sexes and age classes. Front Neurosci. 2016;10:249. 27378834 10.3389/fnins.2016.00249 PMC4908265

[34] De Gregorio C, Zanoli A, Carugati F, Raimondi T, Valente D, Torti V, et al. Parent-offspring turn-taking dynamics influence parents’ song structure and elaboration in a singing primate. Front Ecol Evol. 2022;10:906322.

[35] Ghazanfar AA, Eliades SJ. The neurobiology of primate vocal communication. Curr Opin Neurobiol. 2014;28:128–35. 25062473 10.1016/j.conb.2014.06.015 PMC4177356

[36] Ghazanfar AA, Biazzi RB, Zhang YS. The integrative biology of marmoset monkey vocal learning. Philos Trans R Soc Lond B Biol Sci. 2026;381(1943):20250097. 10.1098/rstb.2025.0097 41641492

[37] MacLarnon AM, Hewitt GP. The evolution of human speech: the role of enhanced breathing control. Am J Phys Anthropol. 1999;109(3):341–63. 10407464 10.1002/(SICI)1096-8644(199907)109:3<341::AID-AJPA5>3.0.CO;2-2

[38] Janik VM, Knörnschild M. Vocal production learning in mammals revisited. Philos Trans Soc Lond B Biol Sci. 1836;2021(376):20200244. 10.1098/rstb.2020.0244 PMC8419569 34482736

[39] Hersh TA, Ravignani A, Whitehead H. Cetaceans are the next frontier for vocal rhythm research. Proc Natl Acad Sci U S A. 2024;121(25):2313093121. 10.1073/pnas.2313093121 PMC11194516 38814875

[40] Janik VM. Cetacean vocal learning and communication. Curr Opin Neurobiol. 2014;28:60–5. 25057816 10.1016/j.conb.2014.06.010

[41] Cook PF, Rouse AA, Sawyer E, Miller K, Berns G. Seal and sea lion brains have evolved to support volitional control of vocal behaviour and learning. Science. 2026;391(6790):1146–50. 41818371 10.1126/science.adx9367

[42] Weiss MN, Ellis S, Croft DP. Diversity and consequences of social network structure in toothed whales. Front Mar Sci. 2021;8:688842.

[43] Cabrera AA, Bérubé M, Lopes XM, Louis M, Oosting T, Rey-Iglesia A, et al. A genetic perspective on cetacean evolution. Annu Rev Ecol Evol Syst. 2021;52(1):131–51.

[44] Paterson RS, Rybczynski N, Kohno N, Maddin HC. A total evidence phylogenetic analysis of pinniped phylogeny and the possibility of parallel evolution within a monophyletic framework. Front Ecol Evol. 2020;7(457):1–16.

[45] Connor RC, Smolker R, Bejder L. Synchrony, social behaviour and alliance affiliation in Indian Ocean bottlenose dolphins, *Tursiops aduncus*. Anim Behav. 2006;72(6):1371–8.

[46] Chereskin E, Allen SJ, Connor RC, Krützen M, King SL. In pop pursuit: social bond strength predicts vocal synchrony during cooperative mate guarding in bottlenose dolphins. Philos Trans R Soc Lond B Biol Sci. 1905;379(1905):20230194. 10.1098/rstb.2023.0194 PMC11391284 38768196

[47] Vance H, Madsen PT, Soto NA, Wisniewska DM, Ladegaard M, Hooker S, et al. Echolocating toothed whales use ultra-fast echo-kinetic responses to track evasive prey. eLife. 2021;10:e68825. 10.7554/eLife.68825 PMC8547948 34696826

[48] Madsen PT, Siebert U, Elemans CPH. Toothed whales use distinct vocal registers for echolocation and communication. Science. 2023;379:928–33. 36862790 10.1126/science.adc9570

[49] Herbst CT, Elemans CPH. Vocal registers expand signal diversity in vertebrate vocal communication. Philos Trans R Soc Lond B Biol Sci. 1923;380(1923):20240006. 10.1098/rstb.2024.0006 PMC11966170 40176520

[50] Sørensen PM, Wisniewska DM, Jensen FH, Johnson M, Teilmann J, Madsen PT. Click communication in wild harbour porpoises (*Phocoena phocoena*). Sci Rep. 2018;8:9702. 29946073 10.1038/s41598-018-28022-8 PMC6018799

[51] Videsen SKA, Raimondi T, Sørensen PM, Pedersen MB, Zimmer WMX, van Geel NCF, et al. Extreme rhythm keeping in long-range slow click communication of sperm whales. Ann N Y Acad Sci. 2026;1559(1):e70289. 42145236 10.1111/nyas.70289 PMC13181709

[52] Oliviera C, Wahlberg M, Silva MA, Johnson M, Antunes R, Wisniewska DM, et al. Sperm whale codas may encode individuality as well as clan identity. J Acoust Soc Am. 2016;139:2860–9. 27250178 10.1121/1.4949478

[53] Watkins WA, Schevill WE. Sperm whale codas. J Acoust Soc Am. 1977;62(6):1485–90.

[54] Cholewiak DM, Sousa-Lima RS, Cerchio S. Humpback whale song hierarchical structure: historical context and discussion of current classification issues. Mar Mamm Sci. 2013;29(3):312–32.

[55] Schneider JN, Mercado IIIE. Characterizing the rhythm and tempo of sound production by singing whales. Bioacoustics. 2019;28(3):239–56.

[56] May-Collado LJ, Agnarsson I, Wartzok D. Phylogenetic review of tonal sound production in whales in relation to sociality. BMC Evol Biol. 2007;7(1):136. 17692128 10.1186/1471-2148-7-136 PMC2000896

[57] Charrier I. Mother–offspring vocal recognition and social system in pinnipeds. In: Aubin T, Mathevon N, editors. Coding strategies in vertebrate acoustic communication. Cham: Springer International Publishing; 2020. p. 231–46.

[58] Reichmuth C, Casey C. Vocal learning in seals, sea lions, and walruses. Curr Opin Neurobiol. 2014;28:66–71. 25042930 10.1016/j.conb.2014.06.011

[59] Cook PF, Rouse A, Wilson M, Reichmuth C. A California sea lion (*Zalophus californianus*) can keep the beat: motor entrainment to rhythmic auditory stimuli in a non vocal mimic. J Comp Psychol. 2013;127(4):412–27. 23544769 10.1037/a0032345

[60] Cook PF, Hood C, Rouse A, Reichmuth C. Sensorimotor synchronization to rhythm in an experienced sea lion rivals that of humans. Sci Rep. 2025;15(1):12125. 40312403 10.1038/s41598-025-95279-1 PMC12045976

[61] Chambers LE, Buck JR, Rogers TL. Leopard seal song patterns have similar predictability to nursery rhymes. Sci Rep. 2025;15(1):26099. 40744953 10.1038/s41598-025-11008-8 PMC12313973

[62] Schusterman RJ, Reichmuth C. Novel sound production through contingency learning in the Pacific walrus (*Odobenus rosmarus divergens*). Anim Cogn. 2008;11(2):319–27. 18038276 10.1007/s10071-007-0120-5

[63] Sanvito S, Galimberti F, Miller EH. Observational evidences of vocal learning in southern elephant seals: a longitudinal study. Ethology. 2007;113(2):137–46.

[64] Reichmuth C, Quihuis D. Social transmission of innovative sound production in walruses (*Odobenus rosmarus*). Aquat Mamm. 2022;48(6):720–3.

[65] Stansbury AL, Janik VM. Formant modification through vocal production learning in gray seals. Curr Biol. 2019;29(13):2244–9. 31231051 10.1016/j.cub.2019.05.071

[66] Ralls K, Fiorelli P, Gish S. Vocalizations and vocal mimicry in captive harbor seals. Phoca vitulina Can J Zool. 1985;63(5):1050–6.

[67] Goncharova M, Jadoul Y, Reichmuth C, Fitch WT, Ravignani A. Vocal tract dynamics shape the formant structure of conditioned vocalizations in a harbor seal. Ann N Y Acad Sci. 2024;1538(1):107–16. 39091036 10.1111/nyas.15189

[68] Raimondi T, D’Orazio F, Di Martino D, et al. Learnt formant modulation via upper vocal tract movements in a marine mammal. Discov Anim. 2026;3:2. 41502830 10.1007/s44338-025-00145-z PMC12769615

[69] Schusterman RJ. Temporal patterning in sea lion barking (*Zalophus californianus*). Behav Biol. 1977;20(3):404–8.

[70] Ravignani A, Fitch WT, Hanke FD, Heinrich T, Hurgitsch B, Kotz SA, et al. What pinnipeds have to say about human speech, music, and the evolution of rhythm. Front Neurosci. 2016;10:274. 27378843 10.3389/fnins.2016.00274 PMC4913109

[71] Jouventi P, Cornet A. The sociobiology of pinnipeds. In: Rosenblatt JS, Hinde RA, Beer C, Busnel MC, editors. Advances in the study of behavior. Academic Press; 1980. p. 121–41.

[72] Stirling I. The evolution of mating systems in pinnipeds. In: Eisenberg JF, Kleiman DG, editors. Advances in the study of mammalian behavior. Stillwater, OK: American Society of Mammalogists; 1983. p. 489–527.

[73] De Roy T, Espinoza ER, Trillmich F. Cooperation and opportunism in Galapagos sea lion hunting for shoaling fish. Ecol Evol. 2021;11(14):9206–16. 34306617 10.1002/ece3.7807 PMC8293783

[74] Reichmuth C, Cook PF. Learning shapes behavioural plasticity and opportunism in sea lions. In: Klimley P, Ainley D, Harvey J. (eds.) Complex behaviour and cognition exhibited by species in the marine environment. Springer Nature. In press.

[75] Reidenberg JS, Laitman JT. Generation of sound in marine mammals. In: Budzynski SM, editor. Handbook of behavioral neuroscience. 19th edition. Elsevier; 2010. p. 451–65.

[76] Cranford TW, Amundin M, Norris KS. Functional morphology and homology in the odontocete nasal complex: Implications for sound generation. J Morphol. 1996;228:223–85. 8622183 10.1002/(SICI)1097-4687(199606)228:3<223::AID-JMOR1>3.0.CO;2-3

[77] Elemans CPH, Jiang W, Jensen MH, Pichler H, Mussman BR, Nattestad J, et al. Evolutionary novelties underlie sound production in baleen whales. Nature. 2024;627(8002):123–9. 38383781 10.1038/s41586-024-07080-1

[78] Tervo OM, Christoffersen MF, Parks SE, Kristensen RM, Madsen PT. Evidence for simultaneous sound production in the bowheadwhale (*Balaena mysticetus*). J Acoust Soc Am. 2011;130(4):2257–62. 21973381 10.1121/1.3628327

[79] Madsen PT, Jensen FH, Carder D, Ridgway S. Dolphin whistles: a functional misnomer revealed by heliox breathing. Biol Lett. 2011;8(2):211–3. 21900314 10.1098/rsbl.2011.0701 PMC3297372

[80] Madsen PT, Lammers M, Wisniewska D, Beedholm K. Nasal sound production in echolocating delphinids (*Tursiops truncatus* and *Pseudorca crassidens*) is dynamic, but unilateral: clicking on the right side and whistling on the left side. J Exp Biol. 2013;216:4091–102. 24133152 10.1242/jeb.091306

[81] Ames AE, Beedholm K, Madsen PT. Lateralized sound production in the beluga whale (*Delphinapterus leucas*.). J Exp Biol. 2020;223:jeb226316 10.1242/jeb.226316 32665444

[82] Jensen FH, Johnson M, Ladegaard M, Wisniewska DM, Madsen PT. Narrow acoustic field of view drives frequency scaling in toothed whale biosonar. Curr Biol. 2018;28(23):3878–85. 30449667 10.1016/j.cub.2018.10.037

[83] Watkins WA, Schevill WE, Ray C. Underwater sounds of *Monodon* (narwhal). J Acoust Soc Am. 1971;49(2B):595–9.

[84] Ford J, Fisher H. Underwater acoustic signals of the narwhal (*Monodon monoceros*). Can J Zool. 2011;56:552–60.

[85] Foskolos I, Soto NA, Madsen PT, Johnson M. Deep-diving pilot whales make cheap, but powerful, echolocation clicks with 50 μL of air. Sci Rep. 2019;9:15720. 31673021 10.1038/s41598-019-51619-6 PMC6823382

[86] Jensen FH, Perez JM, Johnson M, Aguilar Soto N, Madsen PT. Calling under pressure: short-finned pilot whales make social calls during deep foraging dives. Proc Biol Sci. 2011;278:3017–25. 21345867 10.1098/rspb.2010.2604 PMC3158928

[87] Kienle SS, Berta A. The better to eat you with: the comparative feeding morphology of phocid seals (Pinnipedia, Phocidae). J Anat. 2016;228(3):396–413. 26646351 10.1111/joa.12410 PMC5341551

[88] Hocking DP, Park T, Rule JP, Marx FG. Prey capture and processing in fur seals, sea lions and the walrus. In: Ethology and behavioral ecology of otariids and the odobenid. Cham: Springer International Publishing; 2021. p. 101–21.

[89] Boness DJ, Don BW. The evolution of maternal care in pinnipeds: new findings raise questions about the evolution of maternal feeding strategies. Bioscience. 1996;46(9):645–54.

[90] Hughes WR, Reichmuth C, Mulsow JL, Næsbye Larsen O. Source characteristics of the underwater knocking displays of a male Pacific walrus (*Odobenus rosmarus divergens*). J Acoust Soc Am. 2011;129(4):2506–2506.

[91] Larsen ON, Reichmuth C. Walruses produce intense impulse sounds by clap-induced cavitation during breeding displays. R Soc Open Sci. 2021;8(6):210197. 34234955 10.1098/rsos.210197 PMC8242830

[92] Ramirez JM, Tryba AK, Peña F. Pacemaker neurons and neuronal networks: an integrative view. Curr Opin Neurobiol. 2004;14(6):665–74. 15582367 10.1016/j.conb.2004.10.011

[93] Koshiya N, Smith JC. Neuronal pacemaker for breathing visualized in vitro. Nature. 1999;400(6742):360–3. 10432113 10.1038/22540

[94] Arshavsky YI. Locomotor CPG of mammals: the role of pacemaker and network mechanisms. J Neurophysiol. 2025;134(1):50–2. 40423682 10.1152/jn.00208.2025

[95] Kello CT, Orden GC. Soft-assembly of sensorimotor function. Nonlinear Dynamics Psychol Life Sci. 2009;13(1):57. 19061545

[96] Chagnaud BP, Baker R, Bass AH. Vocalization frequency and duration are coded in separate hindbrain nuclei. Nat Commun. 2011;2(1):346. 21673667 10.1038/ncomms1349 PMC3166519

[97] Uhlhaas PJ, Roux F, Rodriguez E, Rotarska-Jagiela A, Singer W. Neural synchrony and the development of cortical networks. Trends Cogn Sci. 2010;14(2):72–80. 20080054 10.1016/j.tics.2009.12.002

[98] Stevens WD, Spreng RN. Resting‐state functional connectivity MRI reveals active processes central to cognition. Wiley Interdiscip Rev Cogn Sci. 2014;5(2):233–45. 26304310 10.1002/wcs.1275

[99] Den Heuvel MP, Pol HEH. Exploring the brain network: a review on resting-state fMRI functional connectivity. Eur Neuropsychopharmacol. 2010;20(8):519–34. 20471808 10.1016/j.euroneuro.2010.03.008

[100] Steriade M. Grouping of brain rhythms in corticothalamic systems. Neuroscience. 2006;137(4):1087–106. 16343791 10.1016/j.neuroscience.2005.10.029

[101] Molinari M, Leggio MG, Thaut MH. The cerebellum and neural networks for rhythmic sensorimotor synchronization in the human brain. Cerebellum. 2007;6(1):18–23. 17366263 10.1080/14734220601142886

[102] Nicolelis MA, Baccala LA, Lin RC, Chapin JK. Sensorimotor encoding by synchronous neural ensemble activity at multiple levels of the somatosensory system. Science. 1995;268(5215):1353–8. 7761855 10.1126/science.7761855

[103] Jürgens U. The neural control of vocalization in mammals: a review. J Voice. 2009;23(1):1–10. 18207362 10.1016/j.jvoice.2007.07.005

[104] Briefer EF. Vocal expression of emotions in mammals: mechanisms of production and evidence. Zool. 2012;288:1–20.

[105] Park J, Choi S, Takatoh J, Zhao S, Harrahill A, Han BX, et al. Brainstem control of vocalization and its coordination with respiration. Science. 2024;383:8081. 10.1126/science.adi8081 PMC11223444 38452069

[106] Jean A. Brain stem control of swallowing: neuronal network and cellular mechanisms. Physiol Rev. 2001;81(2):929–69. 11274347 10.1152/physrev.2001.81.2.929

[107] Taylor EW, Jordan D, Coote JH. Central control of the cardiovascular and respiratory systems and their interactions in vertebrates. Physiol Rev. 1999;79(3):855–916. 10390519 10.1152/physrev.1999.79.3.855

[108] Tyack PL. A taxonomy for vocal learning. Philos Trans R Soc Lond B Biol Sci. 2020;375:20180406. 31735157 10.1098/rstb.2018.0406 PMC6895552

[109] Larson CR. On the relation of PAG neurons to laryngeal and respiratory muscles during vocalization in the monkey. Brain Res. 1991;552(1):77–86. 1913183 10.1016/0006-8993(91)90662-f

[110] Gruber-Dujardin E. Role of the periaqueductal gray in expressing vocalization. In: Budzynski SM, editor. Handbook of behavioral neuroscience. 19th edition. Elsevier; 2010. p. 313–27.

[111] Wilson M, Cook PF. Rhythmic entrainment: why humans want to, fireflies can’t help it, pet birds try, and sea lions have to be bribed. Psychon Bull Rev. 2016;23(6):1647–59. 26920589 10.3758/s13423-016-1013-x

[112] von Cramon D, Jürgens U. The anterior cingulate cortex and the phonatory control in monkey and man. Neurosci Biobehav Rev. 1983;7(3):423–5. 6422356 10.1016/0149-7634(83)90049-0

[113] Bush G, Vogt BA, Holmes J, Dale AM, Greve D, Jenike MA, et al. Dorsal anterior cingulate cortex: a role in reward-based decision making. Proc Natl Acad Sci U S A. 2002;99(1):523–8. 11756669 10.1073/pnas.012470999 PMC117593

[114] Plotnikova A. Gray seals sense blood oxygen levels for safer diving. J Exp Biol. 2025;228(11):jeb250565

[115] McKnight JC, Bønnelycke EM, Balfour S, Milne R, Moss SE, Armstrong HC, et al. Cognitive perception of circulating oxygen in seals is the reason they don’t drown. Science. 2025;387(6740):1276–80. 40112059 10.1126/science.adq4921

[116] Balko JA, Bailey JE. Comparative anesthesia and analgesia– marine mammals. In: Lamont LA, Grimm KA, Robertson S, Love L, Schroeder C, editors. Veterinary anesthesia and analgesia: the sixth edition of Lumb and Jones. 2024. p. 1091–109.

[117] De León MC, Rodríguez DH, Dassis M. Cardiorespiratory patterns of male South American sea lions (*Otaria flavescens*) resting on land. J Comp Physiol B. 2024;194(1):7–19. 38345639 10.1007/s00360-024-01533-9

[118] Lyamin OI, Manger PR, Ridgway SH, Mukhametov LM, Siegel JM. Cetacean sleep: an unusual form of mammalian sleep. Neurosci Biobehav Rev. 2008;32(8):1451–84. 18602158 10.1016/j.neubiorev.2008.05.023 PMC8742503

[119] Galton PM, Shepherd JD. Experimental analysis of perching in the European starling (*Sturnus vulgaris*: Passeriformes; Passeres), and the automatic perching mechanism of birds. J Exp Zool A Ecol Genet Physiol. 2012;317(4):205–15. 22539208 10.1002/jez.1714

[120] Schusterman RJ. Vocal learning in mammals with special emphasis on pinnipeds. In: Oller DK, Griebel U, eds. The evolution of communicative flexibility: complexity, creativity, and adaptability in human and animal communication. Cambridge. MA: MIT Press; 2008. p. 41–70.

[121] Schusterman RJ, Feinstein SH. Shaping and discriminative control of underwater click vocalizations in a California sea lion. Science. 1965;150:1743–4. 5858032 10.1126/science.150.3704.1743

[122] Wild B, Rodden FA, Grodd W, Ruch W. Neural correlates of laughter and humour. Brain. 2003;126(10):2121–38. 12902310 10.1093/brain/awg226

[123] Bylsma LM, Gračanin A, Vingerhoets AJ. The neurobiology of human crying. Clin Auton Res. 2019;29(1):63–73. 29687400 10.1007/s10286-018-0526-y PMC6201288

[124] Ivry RB, Spencer RM, Zelaznik HN, Diedrichsen J. The cerebellum and event timing. Ann N Y Acad Sci. 2002;978(1):302–17. 12582062 10.1111/j.1749-6632.2002.tb07576.x

[125] Kotz SA, Brown RM, Schwartze M. Cortico-striatal circuits and the timing of action and perception. Curr Opin Behav Sci. 2016;8:42–5.

[126] Jin DZ, Fujii N, Graybiel AM. Neural representation of time in cortico-basal ganglia circuits. Proc Natl Acad Sci U S A. 2009;106(45):19156–61. 19850874 10.1073/pnas.0909881106 PMC2776432

[127] Schwartze M, Kotz SA. Timing patterns in the extended basal ganglia system. Adv Exp Med Biol. 2024;1455:275–82. 38918357 10.1007/978-3-031-60183-5_15

[128] Penhune VB, Zatorre RJ. Rhythm and time in the premotor cortex. PLoS Biol. 2019;17(6):e3000293. 31158227 10.1371/journal.pbio.3000293 PMC6564023

[129] Merchant H, Mendoza G, Pérez O, Betancourt A, García-Saldivar P, Prado L. Diverse time encoding strategies within the medial premotor areas of the primate. In: Merchant H, Lafuente V, editors. Neurobiology of interval timing. Cham: Springer International Publishing; 2024. p. 117–40. 10.1007/978-3-031-60183-5_7 38918349

[130] Cook PF, Reichmuth C, Rouse AA, Libby LA, Dennison SE, Carmichael OT, et al. Algal toxin impairs sea lion memory and hippocampal connectivity, with implications for strandings. Science. 2015;350(6267):1545–7. 26668068 10.1126/science.aac5675

[131] Cook PF, Hoard VA, Dolui S, Frederick BD, Redfern R, Dennison SE, et al. An MRI protocol for anatomical and functional evaluation of the California sea lion brain. J Neurosci Methods. 2021;353:109097. 33581216 10.1016/j.jneumeth.2021.109097

[132] Cook PF, Berns GS, Colegrove K, Johnson S, Gulland F. Postmortem DTI reveals altered hippocampal connectivity in wild sea lions diagnosed with chronic toxicosis from algal exposure. J Comp Neurol. 2018;526(2):216–28. 28875534 10.1002/cne.24317

[133] Berns GS, Cook PF, Foxley S, Jbabdi S, Miller KL, Marino L. Diffusion tensor imaging of dolphin brains reveals direct auditory pathway to temporal lobe. Proc R Soc Lond B Biol Sci. 1811;2015(282):20151203. 10.1098/rspb.2015.1203 PMC4528565 26156774

[134] Orekhova K, Selmanovic E, Gasperi R, Gama Sosa MA, Wicinski B, Maloney B. Multimodal assessment of bottlenose dolphin auditory nuclei using 7-Tesla MRI, immunohistochemistry and stereology. Vet Sci. 2022;9(12):692. 10.3390/vetsci9120692 PMC9781543 36548853

[135] Gerussi T, Graïc JM, Peruffo A, Behroozi M, Schlaffke L, Huggenberger S, et al. The prefrontal cortex of the bottlenose dolphin (*Tursiops truncatus* Montagu, 1821): a tractography study and comparison with the human. Brain Struct Funct. 1821;228(8):1963–76. 10.1007/s00429-023-02699-8 PMC10517040 37660322

[136] Flem S, Berns G, Inglis B, Niederhut D, Montie E, Deacon T, et al. Lateralized cerebellar connectivity differentiates auditory pathways in echolocating and non-echolocating whales. PLoS ONE. 2025;20(6):0323617. 10.1371/journal.pone.0323617 PMC12143552 40478798

[137] Ridgway SH. The cetacean central nervous system. In: Comparative neuroscience and neurobiology. Boston, MA: Birkhäuser Boston; 1988. p. 20–5.

[138] Marino L, Rilling JK, Lin SK, Ridgway SH. Relative volume of the cerebellum in dolphins and comparison with anthropoid primates. Brain Behav Evol. 2000;56(4):204–11. 11154999 10.1159/000047205

[139] Ridgway SH, Hanson AC. Sperm whales and killer whales with the largest brains of all toothed whales show extreme differences in cerebellum. Brain Behav Evol. 2014;83(4):266–74. 24852603 10.1159/000360519

[140] Ishikawa T, Tomatsu S, Izawa J, Kakei S. The cerebro-cerebellum: could it be loci of forward models? Neurosci Res. 2016;104:72–9. 26704591 10.1016/j.neures.2015.12.003

[141] Sokolov AA, Miall RC, Ivry RB. The cerebellum: adaptive prediction for movement and cognition. Trends Cogn Sci. 2017;21(5):313–32. 28385461 10.1016/j.tics.2017.02.005 PMC5477675

[142] Beedholm K, Ladegaard M, Madsen PT, Tyack PL. Latencies of click-evoked auditory responses in a harbor porpoise exceed the time interval between subsequent echolocation clicks. J Acoust Soc Am. 2023;153:952–60. 36859123 10.1121/10.0017163

[143] Ridgway SH, Carlin KP, Alstyne KR, Hanson AC, Tarpley RJ. Comparison of dolphins’ body and brain measurements with four other groups of cetaceans reveals great diversity. Brain Behav Evol. 2017;88:235–57. 10.1159/000454797 PMC5348735 28122370

[144] Cook PF, Berns G. Volumetric and connectivity assessment of the caudate nucleus in California sea lions and coyotes. Anim Cogn. 2022;25(5):1231–40. 36114948 10.1007/s10071-022-01685-7

[145] Baer E, Nguyen PD, Lilly S, Song J, Yee M, Matz O, et al. Predictive methods and probabilistic mapping of subcortical brain components in fossil carnivora. J Comp Neurol. 2025;533(1):70014. 10.1002/cne.70014 39786329

[146] Dunbar RI, Shultz S. Evolution in the social brain. Science. 2007;317(5843):1344–7. 17823343 10.1126/science.1145463

[147] Atzil S, Gao W, Fradkin I, Barrett LF. Growing a social brain. Nat Hum Behav. 2018;2(9):624–36. 31346259 10.1038/s41562-018-0384-6

[148] Sakai M, Morisaka T, Kogi K, Hishii T, Kohshima S. Fine-scale analysis of synchronous breathing in wild Indo-Pacific bottlenose dolphins (*Tursiops aduncus*). Behav Processes. 2010;83(1):48–53. 19850113 10.1016/j.beproc.2009.10.001

[149] Mann J, Smuts B. Behavioral development in wild bottlenose dolphin newborns (*Tursiops* sp.) Behavior. 1999;136(5):529–66.

[150] Sakai M, Morisaka T, Iwasaki M, Yoshida Y, Wakabayashi I, Seko A, et al. Mother-calf interactions and social behavior development in Commerson’s dolphins (*Cephalorhynchus commersonii*). J Ethol. 2013;31(3):305–13.

[151] Fellner W, Bauer GB, Stamper SA, Losch BA, Dahood A. The development of synchronous movement by bottlenose dolphins (*Tursiops truncatus*). Mar Mamm Sci. 2013;29(3):203–25.

[152] Huetz C, Saloma A, Adam O, Andrianarimisa A, Charrier I. Ontogeny and synchrony of diving behavior in humpback whale mothers and calves on their breeding ground. J Mammal. 2022;103(3):576–85.

[153] Szabo A, Duffus D. Mother-offspring association in the humpback whale, *Megaptera novaeangliae*: following behaviour in an aquatic mammal. Anim Behav. 2008;75(3):1085–92.

[154] Tyson RB, Friedlaender AS, Ware C, Stimpert AK, Nowacek DP. Synchronous mother and calf foraging behaviour in humpback whales *Megaptera novaeangliae*: insights from multi-sensor suction cup tags. Mar Ecol Prog Ser. 2012;457:209–20.

[155] Noren SR, Edwards EF. Infant position in mother-calf dolphin pairs: formation locomotion with hydrodynamic benefits. Mar Ecol Prog Ser. 2011;424:229–36.

[156] Hartman KL, Harst P, Vilela R. Continuous focal group follows operated by a drone enable analysis of the relation between sociality and position in a group of male Risso’s dolphins (*Grampus griseus*). Front Mar Sci. 2020;7(283):1–13.32802822

[157] Senigaglia V, Whitehead H. Synchronous breathing by pilot whales. Mar Mamm Sci. 2012;28(1):213–9.

[158] Hastie GD, Wilson B, Tufft LH, Thompson PM. Bottlenose dolphins increase breathing synchrony in response to boat traffic. Mar Mamm Sci. 2003;19(1):74–084.

[159] Cioffi WR, Quick NJ, Foley HJ, Waples DM, Swaim ZT, Shearer JM, et al. Adult male Cuvier’s beaked whales (*Ziphius cavirostris*) engage in prolonged bouts of synchronous diving. Mar Mamm Sci. 2021;37(3):1085–100.

[160] Aguilar de Soto N, Visser F, Tyack PL, Alcazar J, Ruxton G, Arranz P, et al. Fear of killer whales drives extreme synchrony in deep diving beaked whales. Sci Rep. 2020;10(1):13. 32029750 10.1038/s41598-019-55911-3 PMC7005263

[161] McCue LM, Cioffi WR, Heithaus MR, Barrè L, Connor RC. Synchrony, leadership, and association in male Indo-pacific bottlenose dolphins (*Tursiops aduncus*). Ethology. 2020;126:741–50.

[162] Chereskin E, Connor RC, Friedman WF, Jensen FH, Allen SJ, Krützen M, et al. Allied male dolphins use vocal exchanges to “bond at a distance.” Curr Biol. 2022;32(7):1657–63. 35334229 10.1016/j.cub.2022.02.019

[163] Moore BL, Connor RC, Allen SJ, Krützen M, King SL. Acoustic coordination by allied male dolphins in a cooperative context. Proc R Soc Lond B Biol Sci. 2020;287:20192944. 10.1098/rspb.2019.2944 PMC7209066 32228413

[164] King SL, Connor RC, Montgomery SH. Social and vocal complexity in bottlenose dolphins. Trends Neurosci. 2022;45(12):881–3. 36404454 10.1016/j.tins.2022.09.006

[165] Benoit-Bird KJ, Au WWL. Cooperative prey herding by the pelagic dolphin, *Stenella longirostris*. J Acoust Soc Am. 2009;125(1):125–37. 19173400 10.1121/1.2967480

[166] Rossbach KA. Cooperative feeding among bottlenose dolphins (*Tursiops truncatus*) near Grand Bahama Island. Bahamas Aquat Mamm. 1999;25:163–7.

[167] Duffy-Echevarria EE, Connor RC, Aubin DJ. Observations of strand-feeding behavior by bottlenose dolphins (*Tursiops truncatus*) in Bull Creek. South Carolina Mar Mamm Sci. 2008;24(1):202–6.

[168] Hoese HD. Dolphin feeding out of water in a salt marsh. J Mammal. 1971;52:222–3. 5101898

[169] Petricig RO. Bottlenose dolphins (*Tursiops truncatus*) in Bull Creek, South Carolina. PhD dissertation. University of Rhode Island; 1995.

[170] Pitman RL, Durban JW. Cooperative hunting behavior, prey selectivity and prey handling by pack ice killer whales (*Orcinus orca*), type B, in Antarctic Peninsula waters. Mar Mamm Sci. 2012;28(1):16–36.

[171] Janik VM, Sayigh LS. Communication in bottlenose dolphins: 50 years of signature whistle research. J Comp Physiol A Neuroethol Sens Neural Behav Physiol. 2013;199(6):479–89. 23649908 10.1007/s00359-013-0817-7

[172] Nakahara F, Miyazaki N. Vocal exchanges of signature whistles in bottlenose dolphins (*Tursiops truncatus*). J Ethol. 2011;29:309–20.

[173] King S, Harley HE, Janik VM. The role of signature whistle matching in bottlenose dolphins (*Tursiops truncatus*). Anim Behav. 2014;96:79–86.

[174] Payne RS, McVay S. Songs of humpback whales. Science. 1971;173(3997):585–97. 17833100 10.1126/science.173.3997.585

[175] Noad MJ, Cato DH, Bryden MM, Jenner MN, Curt K, Jenner S. Cultural revolution in whale songs. Nature. 2000;408:537. 11117730 10.1038/35046199

[176] Garland EC, Goldizen AW, Rekdahl ML, Constantine R, Garrigue C, Hauser ND, et al. Dynamic horizontal cultural transmission of humpback whale song at the ocean basin scale. Curr Biol. 2011;21(8):687–91. 21497089 10.1016/j.cub.2011.03.019

[177] Zwamborn EMJ, Whitehead H. Repeated call sequences and behavioural context in long-finned pilot whales off Cape Breton, Nova Scotia, Canada. Bioacoustics. 2017;26(2):169–83.

[178] Rendell L, Whitehead H. Vocal clans in sperm whales (*Physeter macrocephalus*). Proc R Soc Lond B Biol Sci. 2003;270(1512):225–31. 10.1098/rspb.2002.2239 PMC1691237 12614570

[179] Herzing D. Synchronous and rhythmic vocalizations and correlated underwater behavior of free-ranging Atlantic spotted dolphins (*Stenella frontalis*) and bottlenose dolphins (*Tursiops truncatus*) in the Bahamas. Anim Behav Cogn. 2015;2(1):14–29.

[180] Hersh TA, Gero S, Rendell L, Cantor M, Weilgart L, Amano M, et al. Evidence from sperm whale clans of symbolic marking in non-human cultures. Proc Natl Acad Sci U S A. 2022;119(37):e2201692119. 36074817 10.1073/pnas.2201692119 PMC9478646

[181] Gentry RL. The development of social behavior through play in the Steller sea lion. Am Zool. 1974;14:391–403.

[182] Insley S, Phillips AV, Charrier I. A review of social recognition in pinnipeds. Aquat Mamm. 2003;29(2):181–201.

[183] Sepúlveda M, Harcourt RG. Maternal behavior in otariids and the walrus. In: Campagna C, Harcourt R, editors. Ethology and behavioral ecology of otariids and the odobenid. Cham: Springer International Publishing; 2021. p. 51–61.

[184] Llamazares-Martín C, Palagi E. Playing at the edge of the sea: a comparative analysis in otariids and odobenids. In: Campagna C, Harcourt R, editors. Ethology and behavioral ecology of otariids and the odobenid. Cham: Springer International Publishing; 2021. p. 391–412.

[185] Wilson SC. The role of play in the social development of grey seal (*Halichoerus grypus*) pups with comparative notes on the harbour seal (*Phoca vitulina*). Animals : an open access journal from MDPI. 2024;14(14):2086. 10.3390/ani14142086 PMC11273769 39061549

[186] Páez-Rosas D, Vaca L, Pepolas R. Hunting and cooperative foraging behaviour of Galapagos sea lions. Mar Mamm Sci. 2020;36:386–91.

[187] Hansen MJ, Kurvers RHJM, Licht M, Häge J, Pacher K, Dhellemmes F, et al. California sea lions interfere with striped marlin hunting behaviour in multi-species predator aggregations. Philos Trans R Soc Lond B Biol Sci. 1878;378(1878):20220103. 10.1098/rstb.2022.0103 PMC10107233 37066648

[188] Renouf D. The behaviour of pinnipeds. Springer Science & Business Media; 2012.

[189] Terhune JM. The underwater vocal complexity of seals (Phocidae) is not related to their phylogeny. Can J Zool. 2019;97(3):232–40.

[190] Mathevon N, Casey C, Reichmuth C, Charrier I. Northern elephant seals memorize the rhythm and timbre of their rivals’ voices. Curr Biol. 2017;27(15):2352–6. 28736171 10.1016/j.cub.2017.06.035

[191] Schusterman RJ, Balliet RF. Underwater barking by male sea lions (*Zalophus californianus*). Nature. 1969;222(5199):1179–81. 5788987 10.1038/2221179a0

[192] Peterson RS, Bartholomew GA. Airborne vocal communication in the California sea lion. Zalophus californianus Anim Behav. 1969;17:17–24.

[193] Schusterman RJ, Southall BL, Kastak D, Reichmuth Kastak C. Pinniped vocal communication: form and function. In: Proceedings of the 17th international congress on acoustics. Rome, Italy; 2001. p. 1–2.

[194] Pouw W, Proksch S, Drijvers L, Gamba M, Holler J, Kello C, et al. Multilevel rhythms in multimodal communication. Philos Trans R Soc Lond B Biol Sci. 1835;2021(376):20200334. 10.1098/rstb.2020.0334 PMC8380971 34420378

[195] Connor R, Smolker R. ‘Pop’ goes the dolphin: a vocalization male bottlenose dolphins produce during consortships. Behaviour. 1996;133:643–62.

[196] Vollmer NL, Hayek LAC, Heithaus MR, Connor RC. Further evidence of a context-specific agonistic signal in bottlenose dolphins: the influence of consortships and group size on the pop vocalization. Behavior. 2015;152:1979–2000.

[197] King SL, Allen SJ, Krützen M, Connor RC. Vocal behaviour of allied male dolphins during cooperative mate guarding. Anim Cogn. 2019;22:991–1000. 31317352 10.1007/s10071-019-01290-1 PMC6834747

[198] Hill-Cousins S, Chereskin E, Allen SJ, Connor RC, Krützen M, Papageorgiou D, et al. Allied male dolphins use synchronous displays to strengthen social bonds in a cooperative context. Mov Ecol. 2025;13(1):84. 41272825 10.1186/s40462-025-00603-z PMC12636197

[199] Tripp EA, Fu F, Pauls SD. Evolutionary Kuramoto dynamics. Proc R Soc Lond B Biol Sci. 1986;289(1986):20220999. 10.1098/rspb.2022.0999 PMC9653234 36350204

[200] Jadoul Y, Ravignani A. Modeling the emergence of synchrony from decentralized rhythmic interactions in animal communication. Proc R Soc Lond B Biol Sci. 2003;290(2003):20230876. 10.1098/rspb.2023.0876 PMC10354483 37464759

[201] Lattenkamp EZ, Vernes SC. Vocal learning: a language-relevant trait in need of a broad cross-species approach. Curr Opin Behav Sci. 2018;21:209–15.

[202] Wirthlin ME, Schmid TA, Elie JE, Zhang X, Kowalczyk A, Redlich R, et al. Vocal learning-associated convergent evolution in mammalian proteins and regulatory elements. Science. 2024;383(6690):eabn3263. 38422184 10.1126/science.abn3263 PMC11313673

[203] Price J. Why is birdsong so repetitive? Signal detection and the evolution of avian singing modes. Behaviour. 2013;150:995–1013.

[204] Deoniziak K, Osiejuk TS. Disentangling relations among repertoire size, song rate, signal redundancy and ambient noise level in European songbird. Ethology. 2016;122(9):734–44.

[205] Dunlop RA, Cato DH, Noad MJ. Your attention please: increasing ambient noise levels elicits a change in communication behaviour in humpback whales (*Megaptera novaeangliae*). Proc R Soc Lond B Biol Sci. 2010;277(1693):2521–9. 10.1098/rspb.2009.2319 PMC2894914 20392731

